# Dietary change revealed in kitchen refuse pits from the ancient floors of Housepit 54, K'etxelknáz (Bridge River Site), British Columbia

**DOI:** 10.3389/fnut.2025.1716684

**Published:** 2026-01-29

**Authors:** Anna Marie Prentiss, Ashley Hampton, Jeannie Larmon, Megan Denis, Thomas A. Foor, Haley O'Brien, Nathan Goodale, Matthew J. Walsh, Alysha Edwards, Joshua Jack, Ethan Ryan

**Affiliations:** 1 Department of Anthropology, University of Montana, Missoula, MT, United States; 2 Department of Anthropology, Hamilton College, Clinton, NY, United States; 3 Historical Research Associates Inc., Missoula, MT, United States; 4 National Museum of Denmark, Copenhagen, Denmark; 5 St'át'imc Nation, Lillooet, BC, Canada

**Keywords:** dietary change, Pacific Northwest region, salmon, Bridge River archaeological site, sediment geochemical research, sediment micromorphology research, ancient refuse pits

## Abstract

**Introduction:**

Dietary change in traditional fishing and foraging societies has been examined from standpoints of resource accessibility, population demands, and social needs. Typically, scholars focus on singular models to explain diet choice including those from optimal foraging theory, socio-ecology, and political and historical ecology. It is far less common that we are able to evaluate multiple factors affecting shifting diets and associated cooking procedures within a singular archaeological context.

**Methods:**

In this paper, we draw data from the contents of deep pits filled with kitchen refuse from the 15 stratified anthropogenic floors of Housepit 54, Bridge River Site (K'etxelknáz), British Columbia. We distinguish refuse pits from sequentially re-used cache pits drawing on sediment micromorphology, sediment geochemistry, and general pit contents. Then, focusing on the refuse-filled pits, we develop direct insight into kitchen activities by examining variation in faunal and floral remains and geochemical signatures. Multivariate analysis allows us to recognize patterns of co-associations between faunal remains. Botanical remains and geochemical signatures provide additional support for conclusions regarding food procurement and processing. Temporal change in kitchen regimes is compared to trends in regional climate, local population, and house-level social change to assess alternative explanatory models.

**Results and discussion:**

Results implicate the effects of variation in choice of prey and associated processing and transport procedures as primarily related to population and climate-related foraging pressures.

## 1 Introduction

Diet choice remains a central concern among anthropological archaeologists whether they are interested in the economics of diet breadth (1, 2), its implications for major socio-economic change (3, 4), or its social implications (5–7). There are several prevailing frameworks broadly assumed to drive changes in diet choice within human groups. We identify them as climate and resource structure, demography and predation pressure, and social change and associated expectations. The challenge comes with identifying the most likely prevailing scenario in any given cultural sequence.

**Figure 1:**
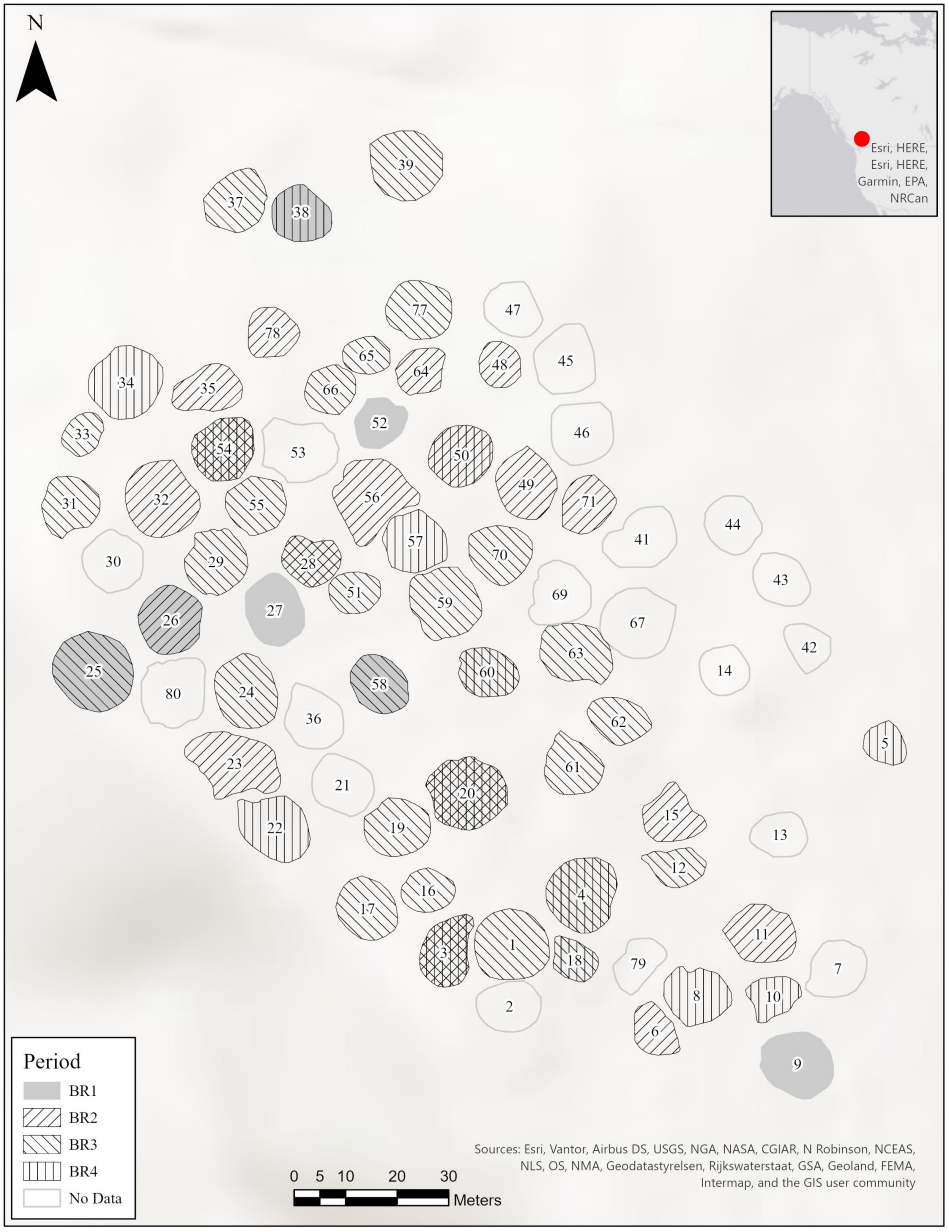
Mid-Fraser region and Bridge River (K'etxelknáz) site map.

**Figure 2:**
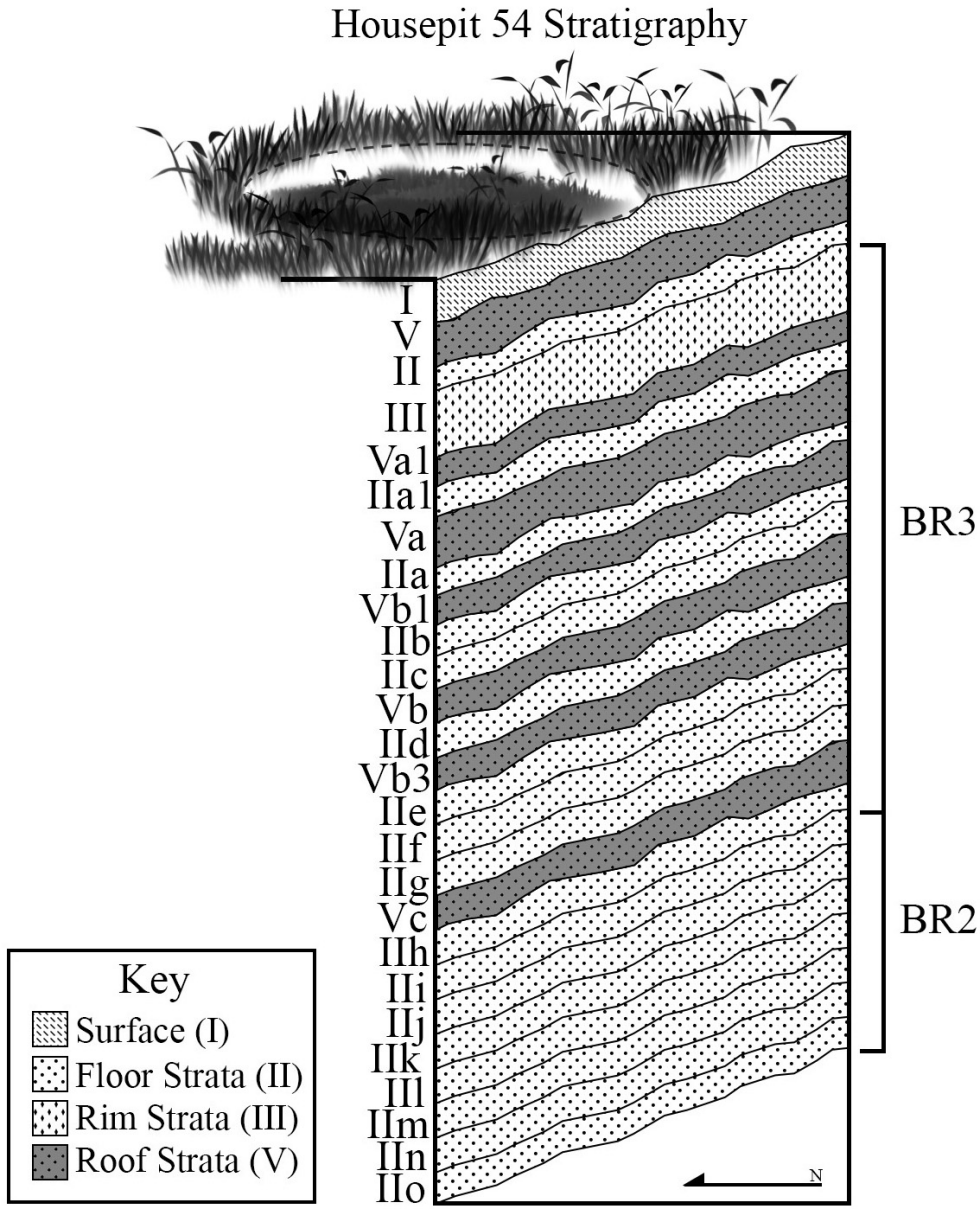
Housepit 54 floor sequence (I = surface, II = floor, V = roof, III = rim midden).

In this study we take advantage of the unique multi-generational occupation record from Housepit 54 at K'etxelknáz, the Bridge River housepit village (Figure 1) in the Mid-Fraser Canyon area of south-central British Columbia to test for evidence of each scenario. Excavations at Housepit 54 revealed a sequence of 15 anthropogenic floors dated to approximately 1,460–1,100 cal. B.P. (8) (Figure 2). Each floor contained multiple features including storage (cache) and kitchen refuse disposal pits (8). While houses elsewhere in the greater Pacific Northwest are known to have multiple floors [i.e., (9)], we know of no excavated house sequence with the same degree of horizontal exposure and high-quality preservation of cultural materials as Housepit 54. The deep floors of Housepit 54 thus provide unprecedented insight into the details of domestic life in an ancient Indigenous village context.

Pit features are common in household contexts across North America's Pacific Northwest region (6, 8, 10), elsewhere in North America [e.g., (11–13)], and around the globe [e.g., (14–17)]. Non-mortuary pits associated within houses take many forms and functions including post-holes, cooking features, storage receptacles, and refuse containers. Defining the functions of pits remains a challenge requiring combined analysis of pit morphology (18, 19), pit-fill stratigraphy (20–22) and contents with regard to artifacts (23, 24) and ecofacts (20, 25). Outcomes of such research can provide significant insights into subsistence strategies (8, 15, 26) and social machinations [e.g., (14, 16, 27–30)].

Refuse disposal pits in the Mid-Fraser villages may contain remains of meals and associated culinary activities and thus, represent ideal contexts for monitoring diet choices within and between occupation floors (6, 8, 31, 32). From a taphonomic perspective, faunal and floral assemblages from refuse pits provide significant advantages over those from open floors. As they represent cleanup of refuse from kitchen contexts, the associated materials would have had less opportunity for taphonomic damage as compared to the high possibility of trampling by foot traffic and ravaging by household dogs (31, 33). Further, deposition in a pit context would have provided a substantially sealed context reducing chances of bio-chemical degradation. If swept/scraped with associated kitchen sediments there also little likelihood of selective bias toward larger items and we can test for that in our geo-chemical studies.

Given the presence of deep refuse pits on most floors, this provides an ideal opportunity to use the contents of these features as well-preserved snap-shots of kitchen activities across the life-span of the house. To accomplish this, we first undertake procedures to separate storage pits from refuse pits. This requires assessment of pit sediments using micromorphology, sediment geo-chemistry, and general contents (artifacts and faunal remains). For the refuse pits, we then assess variation in major element classes of artiodactyls and fish remains. We follow with a review of botanical data and close with a geo-chemical assessment to determine if there might be signatures of foodways as reflected in the faunal and floral evidence.

Prey choice and associated processing, storage, and culinary practices play critical roles in nutrition. As argued by Dent et al. (34), fully understanding ancient nutrition requires significant insights into all aspects of food procurement and processing. They note that at the procurement level, nutrient content in prey species may be affected by soil quality and its effects on plant production. This in turn affects availability and health of associated animal taxa. In the greater Pacific Northwest, geology, elevation, precipitation, hydrology, and latitude combine to create significant ecological zonation with major physio-geographic zones consisting of the Pacific Border, the Coast Ranges, the Fraser-Columbia Plateau, and western Rocky Mountains. Fish, shellfish, plant, avian, reptile, and mammalian resources vary accordingly (35). Local ecosystem productivity is further affected by human manipulation (36–38). Nutrient content of food resources is further affected by harvesting and processing practices (34). In the Mid-Fraser context, there is significant variation in fishing and foraging practices between seasons (39–41) and it is widely known that certain resources (anadromous fish and berries for example) are only available for short time periods (i.e., summer and early fall) whereas other resources may be available to varying degrees year-round [root foods (42)] with some having seasonally variable nutritional characteristics. Artiodactyls for example, will have highest calorie content in mid to late fall (33, 39, 43, 44). Anadromous salmon runs vary by different annual cycles and those cycles may vary significantly and dynamically depending on prevailing ecological and climatic conditions (45).

Food preparation of course impacts bioavailability of many nutrients as well. It is not surprising that many important food resources were processed in raw form for storage thus retaining maximum nutrient profiles. Examples include dried deer and salmon meat along with berries (33, 39, 42, 46). Teit [(40), p. 236] notes that these were often used in combination, whether to improve taste, texture, or nutritional value, or any combination thereof. Other foods required cooking to improve digestibility (many root foods) and to prepare for long term storage (smoked and wind-dried meats) (33, 39, 42). Finally, cooking practices would also to be expected to affect nutritional quality of foods (34). Cooking in and around Mid-Fraser winter houses included pit-roasts of fish and artiodactyls; open hearth roasting of meat; and creation of stews combining fish, mammal meat, berries, and other plant contributions. In this study, we do not attempt to formally model nutritional variability but rather take an important step in the direction by assessing food transport and associated culinary decision-making with regards to contributions of artiodactyl, salmonid, and berry species in the contexts of constraints and opportunities potentially imposed by variability in climate conditions, local populations, and social change.

Results suggest that managers of traditional kitchens of Housepit 54 adapted not just their procurement and transport decisions, but also their culinary practices to align with both constraints and opportunities driven largely by population pressures but also climate factors. Both artiodactyls and salmonids were differentially processed and transported not in isolation but in relation to costs and benefits of the other food source. Thus, highly abundant local artiodactyls could trigger more selective transport of high utility salmon sections. In contrast, sparse local artiodactyls favored less selectivity in salmon parts, but greater selectivity in artiodactyl elements. Drawing from currently limited data it would appear that berry harvest from local plants occurred most frequently when local artiodactyl hunting was the dominant pattern.

## 2 Research problem: three alternative explanatory scenarios

Our fundamental goal here is to better understand the conditioners of shifts in culinary behavior across the floors of Housepit 54. In turn we hope the study contributes toward general theorizing of diet choice and food processing behavior and its effects on nutrition in human populations. Toward that end, we review three major models (climate, population, socio-cultural) with a particular focus on the Pacific Northwest region.

Climate variation is widely recognized as a significant factor in the structure of prey populations and consequently variability in human diet options. An extensive literature exists on the effects of climate on productivity of marine and anadromous fish species in the North Pacific [i.e., (47–51)]. Likewise, there has been substantial research into climate variability and its effects on terrestrial ecosystems and their impacts on game populations and economically important plant species (especially berries) in the greater Pacific Northwest [i.e., (52–55)]. In general, mesic (cooler and wetter) periods favor stronger fisheries and with that higher salmon production (56–59). Climate impacts on mammal populations are complex with multiple variables affecting outcomes. However, a variety of studies suggest that in western North America cold/snowy winters coupled with low moisture summers adversely impact artiodactyl populations and in contrast, warmer and drier winters and wetter summers favor population growth (60–64). Finally, there is an extensive record of climate and vegetation change across the Pacific Northwest region [i.e., (65–69)]. As noted by Turner (70) this in turn had a variety of effects on distributions of edible and technologically valuable plants around the region, for example with fire regimes most common in warmer and drier periods positively favoring production of berry producing plants during initial re-growth periods (55).

While scholars universally recognize the impacts of climate change on productivity of marine, riverine, lacustrine, and terrestrial taxa, it is also clear that human actions have impacts. Human groups may modify habitats to enhance productivity as associated with salmon spawning grounds (38), shallow-water clam production (36), and careful use of fire to favor growth (and rapid recovery) of particular plant species and to attract favored animal taxa (37). In contrast there is also abundant evidence that human predation pressure and practices can have adverse effects on prey populations. Broughton (71, 72) demonstrates the impact of rising human populations on a range of prey in the Sacramento River valley area. Cole et al. (73) provide evidence for human settlement density and associated predation-pressure impacting species abundance in northeast California. Localized impacts of prey intensification have been demonstrated in the middle Columbia (48), in the Mid-Fraser (74, 75), and across the wider Pacific Northwest (76). Beyond prey species, plants and landscapes may also be impacted. While it is hard to over-harvest berry species, it is possible that select geophyte taxa may have been over-harvested at times (70, 74). Impacts of these demographically-driven scenarios on human subsistence decisions usually include expanding diet breadth, extended processing of lower utility prey species, and in some contexts, extensified foraging across landscapes (72, 74, 77).

A third set of factors conditioning diet depart from demo-ecological concerns and raise the possibility of socio-culturally constructed preferences. As noted by Russell (7), this could take several forms. First, household diet could simply be affected by having particularly effective members. Romanoff (43) notes that Mid-Fraser domestic groups always ate better when an outstanding hunter was part of the household. He notes however, that this circumstance could provide opportunities for enhanced social standing for the household via generosity to others. Taking such behavior to its logical extreme we recognize feasting and competitive generosity in some groups. As summarized by Perodie (78) and Hayden (5, 6), there were many kinds of feasts across the Pacific Northwest region including solidarity, reciprocal, solicitation, promotional, competitive, political support, acquisition of political position, work party feasts, and child-growth feasts. While each had its distinctions with regard to quantities, and types of food along with preparation and social contexts, a commonalty of feasting in general is higher quantities than usual food cooking consumption events, and in some cases (competitive and political) rare and/or expensive items considered delicacies being included (5–7). In the Pacific Northwest this might include eulachon, salmon, and in some contexts, even whale oil; special meat (select cuts from artiodactyl species and canids in some contexts); and/or use of local favorite recipes (fish-head soup in the Mid-Fraser, for example). Ames (79) argues that gifting and feasting complexes were critical components of strategies designed to prevent demographic crises for long-lived competitive house groups. Demonstration of economic success via displays and generosity (as for example with potlatching) attracted membership within and beyond kin groups. Prentiss (35) suggests that these concerns were widespread among many Pacific Northwest groups organized around House-level socio-political units.

## 3 Materials and methods

### 3.1 . Defining data source and assessing pit types

Our first challenge is to distinguish between storage and refuse pits. Here we introduce Mid-Fraser storage and refuse pits and review testing procedures focused on pit stratigraphy and fill contents. Geochemical measures are important at this stage though we review specific methods below under Pit Contents.

#### 3.1.1 . Defining data source: cache pits and refuse pits

We draw our data for this study from the fill of wide and deep pits from the floors of Housepit 54. These features, often termed cache pits, may have had multiple functions. The traditional St'át'imc food storage system could be complex with food moved between storage features in three contexts. Procurement sites, whether canyon-bottom fish camps or upland hunting and geophyte processing camps, could include both below- and above ground storage features (33, 39). Below ground features could be nearly two meters deep and nearly as wide. They were lined with birch bark and sometimes layered with pine straw to repel insects and moisture [(33, 40), p. 234]. Similarly designed and sized cache pits could also be placed external to houses within pithouse villages. Finally, indoor cache pits were also established at often smaller sizes—at the K'etxelknáz (Bridge River site) village they tend to be in the range of 50–150 cm wide and deep, though there are occasional larger exceptions. Dried and often frozen food items such as *tswan* (dried salmon) and artiodactyl meat were moved between field, village and house storage features depending upon need and planned use. Field storage contexts could also serve as backups in case of unexpected loss within the village facilities. House storage pits were generally for short-term storage. Thus, some household pits could theoretically have been used and reused multiple times with proper cleaning and refurbishing in between use episodes. However, it is also quite likely that many household pits were simply used briefly for storage and/or converted for use as refuse receptacles.

#### 3.1.2 . Testing for pit type

Deep pits used for refuse disposal provide potentially unique insights into kitchen activities as compared to scattered open floor materials. Pit contents likely represent rapid cleanup across multiple occasions and sealing within pits, thus preventing further mechanical destruction of bone. Chemical preservation conditions are apparently very good at Housepit 54 as excavators often commented on the “fresh” conditions of many bones and also the decay-related odor emanating from many pits, those used in this study pre-dating 1,100 years ago. We assess variation in pits using three approaches. First, we examine the general sediment content, visual evidence for bedding, and frequencies of cultural items including faunal remains, lithic artifacts, and fire-cracked rock. We expect bedded pits to also contain fewer cultural items representing their primary use as cache pits. In contrast pits with homogenous sediment fill and more dense cultural items more likely represent kitchen refuse pits. Second, we examine sediment content in greater depth with a micromorphological assessment. This provides more detailed insight into sediment formation processes. Third, we compare geo-chemical and isotopic data between pits to determine if there are measurable differences in organic compounds. Cache pits with little kitchen refuse should have weaker isotopic and geo-chemical signatures than those filled with kitchen refuse. We then determine which were likely rapid fill refuse pits and which where long-term use cache pits.

#### 3.1.3 . Micromorphology

Micromorphological samples were collected during the 2022 field season by the field crew, who carefully documented the context, including photographing and drawing the pit profiles. Samples were taken in the field as intact blocks of various dimensions and wrapped with aluminum foil and packing tape, with their orientation clearly marked. These blocks were then transported to the facilities at Wagner Petrographic in Lindon, Utah. The samples were impregnated with blue epoxy to highlight the voids, sectioned into c. 25–30 μm thin sections, and mounted on 3 × 5 inch slides. Thin sections were examined with binocular and petrographic microscopes in plane-polarized (PPL) and cross-polarized (XPL) light. The analysis follows procedures and language outlined by Stoops (80).

For consideration in this analysis, samples were extracted from two pit contexts, Feature B14 and C5. Feature B14, in floor IIe, is a deep, bell-shaped pit. Feature C5, from IIk, is a deep cylindrical pit. The investigation of microstructures and contents of the two different contexts, one of which is visually bedded (B14) and the other of which appears more homogenous (C5) will provides insights into the differential uses of those features.

### 3.2 . Analyzing pit contents

We apply three approaches to measuring variability in kitchen culinary regimes using data from refuse pits between the floors of Housepit 54. This involves studies of faunal remains, paleoethnobotanical materials, and sediment geo-chemical signatures.

#### 3.2.1 . Faunal measures

Previous study of Housepit 54 faunal remains focused primarily on taxonomic representation across the IIl through IIa floors (75). Salmon remains are dominant across all floors and we recognize peaks in salmon densities on the IIe and IIb floors. Artiodactyls are densest on the IIg-IIe floors. The richness measure is of limited value given generally low richness scores across all floors. The evenness measure is approximately inverse to salmon densities. Thus, we recognize greatest evenness on IIh and IIi, IId, and IIa. Floors IIi and IId are recognized as times of population reduction thus the high evenness/low salmon pattern is thought to reflect some degree of subsistence stress (75). To explore this conclusion further, Prentiss et al. (75) graphed abundance indices for mammalian appendicular parts and salmon thoracic vertebrae. The results were intriguing as the single high point for thoracic vertebrae came at the end of a period of low appendicular abundances on IIg and IIf. High appendicular abundance occurs early and late in the sequence, both associated with low thoracic abundances. This suggested to Prentiss et al. that the latter pattern may have coincided with the effects of high local predation pressure on mammals during middle to early-late BR2 (BR2 spans ca. 1,600–1,300 cal. BP) and early-middle BR3 (BR3 spans ca. 1,300–1,000 cal BP) times thus requiring longer hunting trips and necessitating importantly, more complete fish (rather than primarily the fatty parts [thoracic sections]). These results imply that kitchen practices at Housepit 54 may have been driven by variation in access to these two main prey groups (artiodactyls and salmonids). We explore this further in our current study.

Here we quantify fauna drawing on elements identifiable to element class and taxon (at least to the family level) across the refuse pits. Thus, we recognize two main taxonomic groups (Artiodactyla and Salmonidae) and four major anatomical groupings for each. For artiodactyls we subdivide elements into cranial, axial (vertebrae, ribs, innominate, scapula), and appendicular assuming that field transport decisions generally meant separating limbs from all other parts based on relative economic utility and ease of transport (81). This is known from St'át'imc ethnography (33, 39, 82) and is recognized in many other contexts (72, 83). For salmonids we sorted elements into cranial, thoracic, and caudal recognizing that each section has different meat/fat utilities that could influence decisions for transport and cooking (75, 84). All element data were quantified using abundance indices (71, 85). We also quantified overall artiodactyl and salmonid densities. We subjected our data matrix to a principal components analysis (PCA) using SPSS v. 28 with the goal of recognizing significant co-associations between element groups from each taxonomic set and plotting those against stratigraphic context. If the previous analysis of fauna from the overall floors (75) is replicated in the refuse pits data, then it is possible that we may recognize at least two major kitchen patterns: complete artiodactyl/thoracic-dominated salmonid and appendicular dominated artiodactyl/complete salmonid. In turn this may have implications for how household residents managed household nutrition given different animal food combinations.

#### 3.2.2 . Macrobotanical measures

The macrobotanical samples were collected from pit fill sediments and separated from the sediment using a bucket flotation system. The sediment volume was measured, flooded with water and stirred, then the lighter fraction—primarily the botanical material with some lighter fauna—poured off into a screen and dried. The resulting botanical material was sorted via a series of screens (4.0, 2.0, 1.0, and 0.425 mm) and weighed, then examined under a compound microscope and compared to a variety of comparative materials. Only charred material was counted and incomplete specimens counted as half. The material was identified to the species level whenever possible but otherwise at the genus level.

#### 3.2.3 . Geochemistry measures

Sediment samples from pit features were collected systematically based on the spatial extent (number of quads) and stratigraphic depth (number of levels) of each feature. Approximately 250 g of sediment was sieved to < 4 mm to remove coarse material, and the finer fraction was retained for isotopic and elemental analyses. Detailed protocols for sediment preparation, analytical instrumentation, and calibration procedures are provided in Supplementary Materials A.

Stable isotope ratios of carbon (δ^13^C ‰) and nitrogen (δ^15^N ‰) were determined by isotope ratio mass spectrometry (IRMS) to evaluate potential variation in organic input among pit fills. Statistical comparisons of isotopic data were conducted using MANOVA with Tukey's HSD *post hoc* tests and non-parametric Kruskal–Wallis ANOVA with Bonferroni corrections (α = 0.05). An isotope mixing model (SIMMR) was applied to estimate proportional contributions of faunal sources to each feature fill.

Elemental composition was determined by energy-dispersive X-ray fluorescence (ED-XRF) using a Thermo ARL Perform'X spectrometer. Major oxides and trace elements were quantified, with emphasis on Ca, K, and P as these elements are commonly associated with food preparation, midden, and hearth activities [84, p. 5, 6]. Following the approach of Scott (86), elemental concentrations were normalized to Ti to account for lithogenic variation. Normalized element ratios were compared across features using Kruskal–Wallis ANOVA with Bonferroni adjustment (α = 0.05). All statistical analyses were performed in SPSS v29 and RStudio 2025.05.1 (R v4.5.1).

### 3.3 . Models of climate, population, and social change

**Figure 3:**
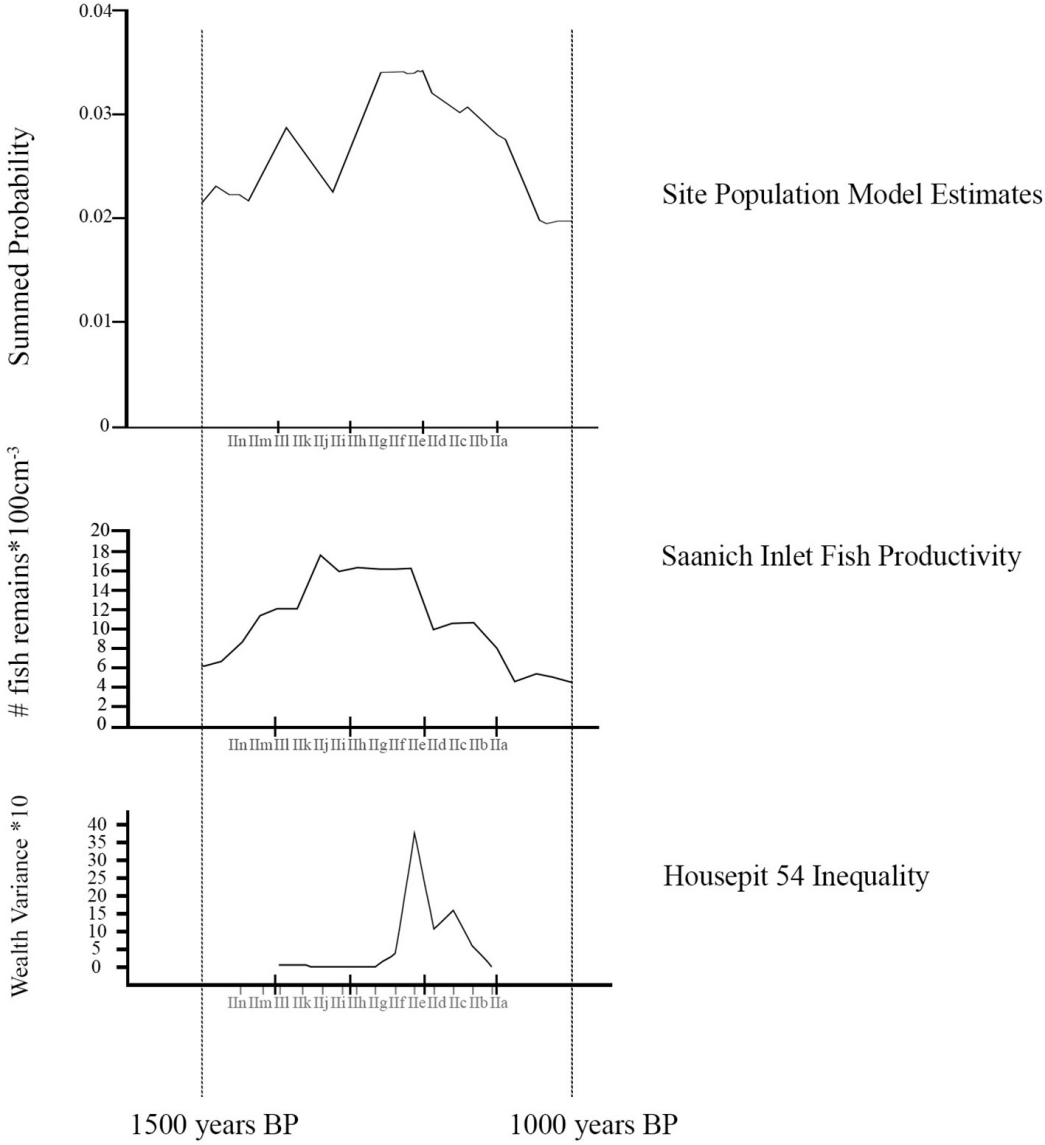
Models of variation in Mid-Fraser Canyon population dynamics [data and modelling code from Prentiss et al. (89)], regional climate [data from Tunnicliffe et al. (59)], and Housepit 54 inequality [data from Prentiss et al. (87)] superimposed on the Housepit 54 floor sequence (IIa to IIn). The Saanich Inlet fish data distribution post-1800 cal. BP is structured by three radiocarbon dated layers [see Tunnicliffe et al. (59)] and an extrapolated date sequence for the other strata. We use this as a heuristic illustration of a general pattern of likely cooler climate conditions and productive fish populations that appears to peak in the general range of ca. 1400-1200 cal. B.P. [see also Patterson et al. (58)].

Once we developed conclusions on kitchen culinary regimes identified for individual floors, we then compared the dating of those floors to models of climate, population variation, and social inequality distributed within the same time range (Figure 3). It is not easy to find a single measure to track cumulative change in any global region. However, given the fundamental importance of the fishery and recognized salmonid sensitivity to climate change as reflected in changed sea-surface temperatures and stream temperatures and sediment bed-load (88), we suggest that general marine productivity is a useful proxy for climate change. In Figure 3, we rely in Tunnicliffe et al.'s [(59), Figure 5] distribution of fish remains from two cores at Saanich Inlet, British Columbia. While not directly a measure of salmon numbers this does provide robust insight into marine productivity across the late Holocene that is reflected in other data sets from other cores, for example, at Effingham Inlet [i.e., (58)].

We measure population on a level specific to the Mid-Fraser Canyon based upon the summed probability model previously developed from radiocarbon dates in Prentiss et al. (89). This provides us insights into approximate population dynamics though not absolute population counts. Finally, we model emergent social inequality specific to Housepit 54 using the wealth variance model of Prentiss et al. (87). Here wealth is measured on the basis of variance in representation of five measures of inequality (non-local raw material, prestige raw material, prestige objects, hunting-related artifacts, and mammalian remains) between activity areas across floors (IIl to IIa) as derived from a PCA.

### 3.4 . Test expectations

We review test expectations associated with the three models for the different data sets: artiodactyl and salmonid procurement and processing as indicated by faunal remains; berry gathering as marked my macrobotanical remains; and geo-chemical signatures deriving from deposition of food remains.

#### 3.4.1 . Test expectations: artiodactyl and salmonid procurement and processing

If the climate model as measured by fisheries productivity is correct, we expect steady increase in access to salmon through the period of ca. 1,600–1,200 cal. B.P., and subsequent rapid decline and instability. The implication here is that diet would progressively narrow as salmon increases in importance leading up to ca. 1,200–1,250 cal. BP and then broaden thereafter. In reference to salmonid anatomical elements, we would expect complete element representation of salmonid elements early (IIn-IIg floors, see Figures 2, 3) shifting to more frequent highest utility (thoracic) sections during optimal conditions (IIf-IId) and back to all elements late (IIc-IIa). As salmonid procurement became dominant, pressure on local artiodactyl populations would decrease. Thus, we would expect more axial artiodactyl parts during peak salmon (IIf-IId) and more appendicular for early (pre-IIf) and late (IIc-IIa).

The population model implicates cycles of demographic boom and bust with subsequent food procurement and processing implications (Figure 3). In this scenario, population changes would have immediate effects on artiodactyl populations. Periods of high pressure on local artiodactyl populations would force hunters to travel farther afield and engage in more intensive field processing. In contrast, for short timespans post-dating human population lows we would expect local rebound in artiodactyl populations thus reducing the need to travel far from the village to procure game. Consequently, we would expect the strongest axial dominated pattern on the IIh to IIf floors and more of an appendicular pattern earlier (IIn-IIi) and later (IIe-IIa). Access to salmonids would not be directly affected by population dynamics given that salmon probably could not be overharvested with traditional technology. However, differential access to artiodactyls could have implications on effort to be expended in procuring and transporting salmonids. Thus, we might expect more high utility salmonid parts (thoracic sections) during times of peak artiodactyl abundance (IIh-IIf) as it would not necessarily be worth the effort to bring entire cranial and vertebral sections from fish home during this time. At other times when hunting was most costly (IIn-IIh and IIe-IIa), we would expect investment in transport of total body fish to be more worthwhile.

The third scenario creates expectations based on social strategizing by house occupants (Figure 3). In this scenario we would expect investment in transport of highest utility artiodactyl and salmonid element sets as associated with amassing quality foods for signaling to other groups as to the socio-economic and demographic success of the Housepit 54 household groups. This kind of strategizing would be typical of the time associated with inter-household inequality, especially during its cooperative phase (IIe) and to a lesser degree its coercive phase (IId-IIb) (90). Thus, we would expect the strongest house-wide signal consisting of a high utility (thoracic-dominated) fish signature and an axial-focused artiodactyl signature to come during IIe and to a lesser degree on subsequent floors (IId-IIb). All other times would be characterized by total fish and appendicular artiodactyl patterns.

#### 3.4.2 . Test expectations: botanicals

Using data from the Tl'atl'lh (Keatley Creek) site, Prentiss et al. (74) demonstrated that during times of wider landscape search for artiodactyls, berry species from higher elevation contexts in the Mid-Fraser area became more common. Thus, in reference to the Housepit 54 sequence, periods of higher appendicular signatures should also produce more frequent berry seeds associated with wetter sub-alpine and alpine environments. Beyond that, ethnographies identify select berry taxa as famine food and higher abundances of those items should be associated with floors characterized by significantly raised costs of procuring game and fish resources. Under the climate scenario this would mean times of weakest marine productivity (IIn to IIg and IIc-IIa) whereas under the population model this would mean during IIn-IIi and IIe-IIa (8, 75, 90) (see Figure 3).

#### 3.4.3 . Test expectations: isotopes and geochemical elements

Prey choice and processing/transport strategies as reflected in kitchen refuse pit sediments can be measured in sediment geo-chemistry, though may be confounded by additional variables. Isotope values reflect pit fill composed of not just kitchen refuse but sediment impacted by other factors such as intensity of floor use, variability of floor activity, cleaning patterns/practices, and population density. Longer occupations over the winters of a floor's history would lead to higher intensity of activities and higher accumulations of isotopes and element values. Variability in floor activity, such as cooking location compared to pit location, types of subsistence items cooked per hearth, and additional activities occurring around these hearths would equally impact pit fill values. Similarly, if there are multiple pits used for refuse then cleanup practices may dictate variability in fill with each pit capturing only nearby cooking activities compared to a singular refuse pit for a floor capturing the entirety of floor activity.

Amidst such variability however, pit fill is expected to reflect consistent soil N cycle processes which would influence final δ15N isotope values (91). Almost all pits are dug into previously anthropogenically altered floor contexts and nearly all pits lack internal structure (micro-bedding) leading to the probability that they represent similar filling-in processes. All pits are located within the pithouse structure and thus influenced by similar repetitive anthropogenic interactions with the floor sediments. Such similarities allow for the assumption that δ13C and δ15N isotope variation per pit fill may be more reflective of overall variation in subsistence consumption/disposal practices. When comparing isotope signatures between pit fill based on food source, if sources included primarily mammals, then the overall δ15N values would be lower while if primarily fish then δ15N values would be higher. For δ13C values, if primarily mammalian taxa then there should be lower concentrations while fish would have higher concentrations. Plant consumption patterns would also potentially impact δ13C values, with C3 plants producing δ13C values around −27‰ in soil organic matter (92).

A majority of major and minor elements can act as proxies for a multitude of different kinds of human activity patterns and oftentimes overlap in the kinds of activities that leave behind enrichment of similar elements (93, 94). Thus, distinguishing between fish or mammal preparation based solely on elemental composition within pit feature fill is not possible. However, by comparing a suite of elements, it can reveal different degrees of elemental enrichment which can highlight if the refuse in a pit feature is distinctive from other pits. For instance, if all elemental markers are much lower, then the fill may be showing lower degrees of intensity/lower population density from the floor activity from which the pit fill was swept. Element comparisons also help highlight potential variances in cleaning practices and refuse deposit processes which may then be helpful for interpretating isotope values.

Aligning these concerns with test expectations discussed above (see also Figure 3), we would expect the strongest fish signatures at times of either peak fisheries productivity under the climate model (IIf-IId) or at times of most costly artiodactyl hunting under the population model (IIn-IIi and IIe-IIa). Artiodactyl signals would be strongest during weaker fisheries production under the climate model (IIn-IIg and IIc-IIa) and at the beginnings of population rebounds after lows under the population model (IIh-IIf). Under the social change model, we would not necessarily expect major change in fish signatures but mammal signatures would be strongest on IIe-IIb given knowledge of emergent social inequality at that time (87).

## 4 Results

### 4.1 . Pit type

We draw upon pit form and content, micromorphology, and sediment geochemistry to distinguish between likely storage vs. kitchen refuse pits.

#### 4.1.1 . Pit type: macroscopic pattern

**Figure 4:**
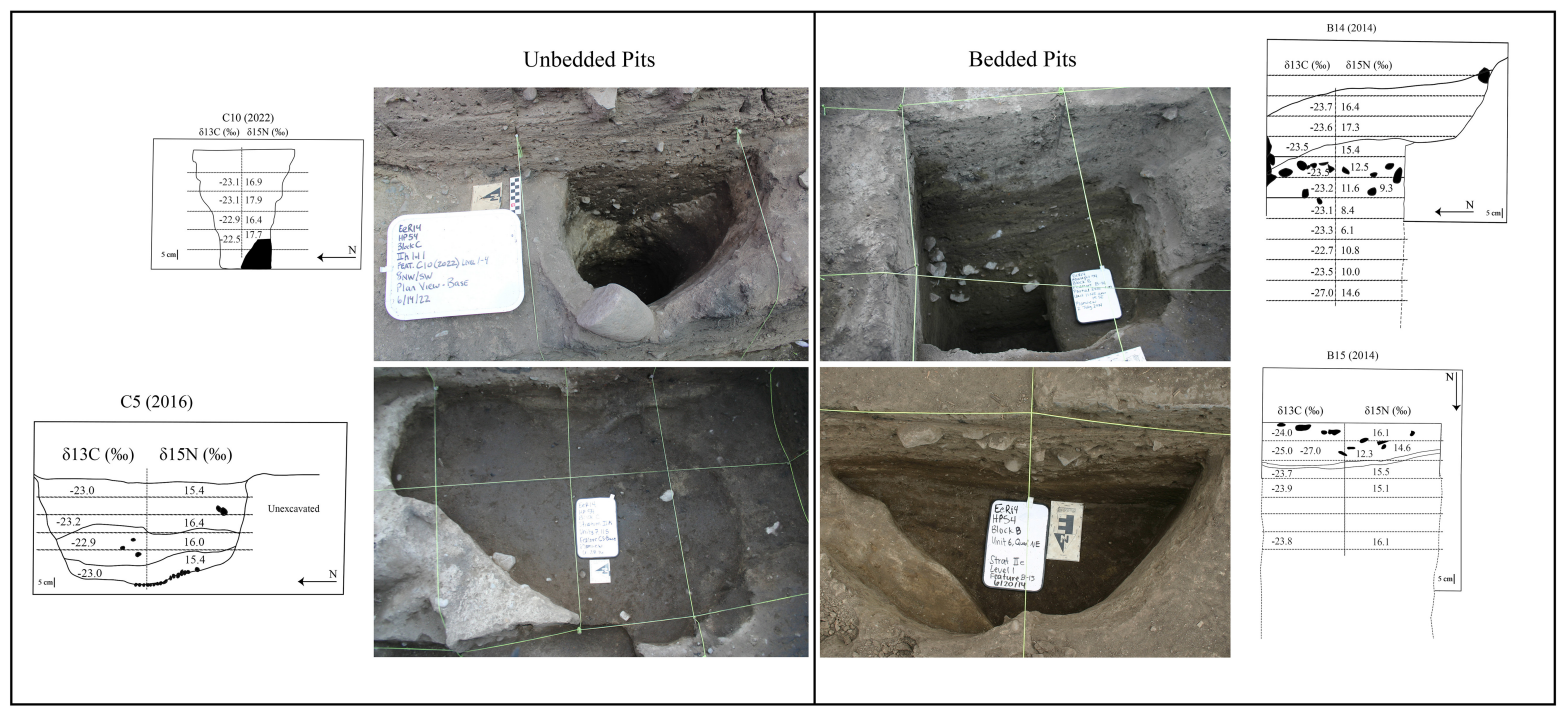
Photographs of select cache pits. Bedding layers in B14 and B15 are macroscopically visible.

We identified 16 large bell-shaped or cylindrical pits with potential for further analysis (Table 1, Figure 4, Supplementary Materials B). With one exception all of the pits from floors IIk to IIc are bell-shaped, while beginning at IIh the deeper floors are primarily cylinder shaped. There is also a distinction in estimated clay content [field estimations following Fladmark (95)] within feature fill. Beginning on IIk, deeper floors are generally over 55% clay while shallower features fall in the 22–53% range. This aligns with general floor composition where the deeper floors are always highest in clay content (32). The implication is that feature fill probably derives from associated floor sediments. Sediments in all but three features are homogeneous without obvious bedding layers. Exceptions include B14 and B15 and A1. The former two have thin bedding lines throughout much of their sedimentary sequence whereas A1 is characterized by fewer and thicker layers. Fire-cracked rock counts from each feature include all those in the pebble and cobble size ranges on the Wentworth scale. Lowest scores are found in D11, B14 and 15, A1 and C5. Feature B15 is exceptionally low. Lithic tool densities are by far the lowest in B14 and B15. Faunal densities are exceptionally low in B15 and to a lesser degree in A17 and A1. Considering all data and the general fill pattern, it would appear that features B14, B15 and A1 not only contain bedded sediments but also lower FCR, lithic tool, and faunal densities.

**Table 1:** Features used in this study (DBSP=Deep Bell-Shaped Pit, DCP=Deep Cylindrical Pit, Vol=Volume, FCR=Fire Cracked Rock, Den=Density).

**Feature (Year)**	**Floor**	**Type**	**Clay**	**Vol./**	**FCR**	**Lithic**	**Faunal**	**Bedded**
			**Percentage**	**1000**	**Den**.	**Den**.	**Den**.	
D8 (2014)	IIc	DBSP	32	212	1.73	0.04	0.98	N
D16 (2016)	IId	DBSP	47	250	1.44	0.14	1.7	N
D20 (2016)	IIe	DBSP	53	251	1.57	0.05	1.54	N
D11 (2016)	IIe	DBSP	49	231	0.14	0.06	0.53	N
B14 (2014)	IIe	DBSP	45	525	0.33	0.006	0.27	Y
B15 (2014)	IIe	DBSP	29	201	0.04	0.005	0.005	Y
B3 (2014)	IIe	DBSP	47	114	4	0.1	0.32	N
A17 (2013)	IIf	DBSP	22	301	1.88	0.02	0.07	N
A1 (2014)	IIg	DBSP	27	216	0.32	0.05	0.14	Y
A5 (2014)	IIh	DBSP	25	376	1.97	0.08	0.86	N
C10 (2022)	IIh	DCP	49	211	0.56	0.03	2.58	N
C5 (2016)	IIk	DBSP	68	589	0.26	0.03	1.15	N
A11 (2016)	IIl	DCP	70	127	1.98	0.06	0.79	N
A14 (2016)	IIm	DCP	49	81	1.73	0.25	2.63	N
A12 (2016)	IIm	DCP	55	94	0.93	0.1	0.66	N
A17 (2016)	IIn	DCP	71	29	0.62	0.28	2.69	N

#### 4.1.2 . Pit type: micromorphology

**Figure 5:**
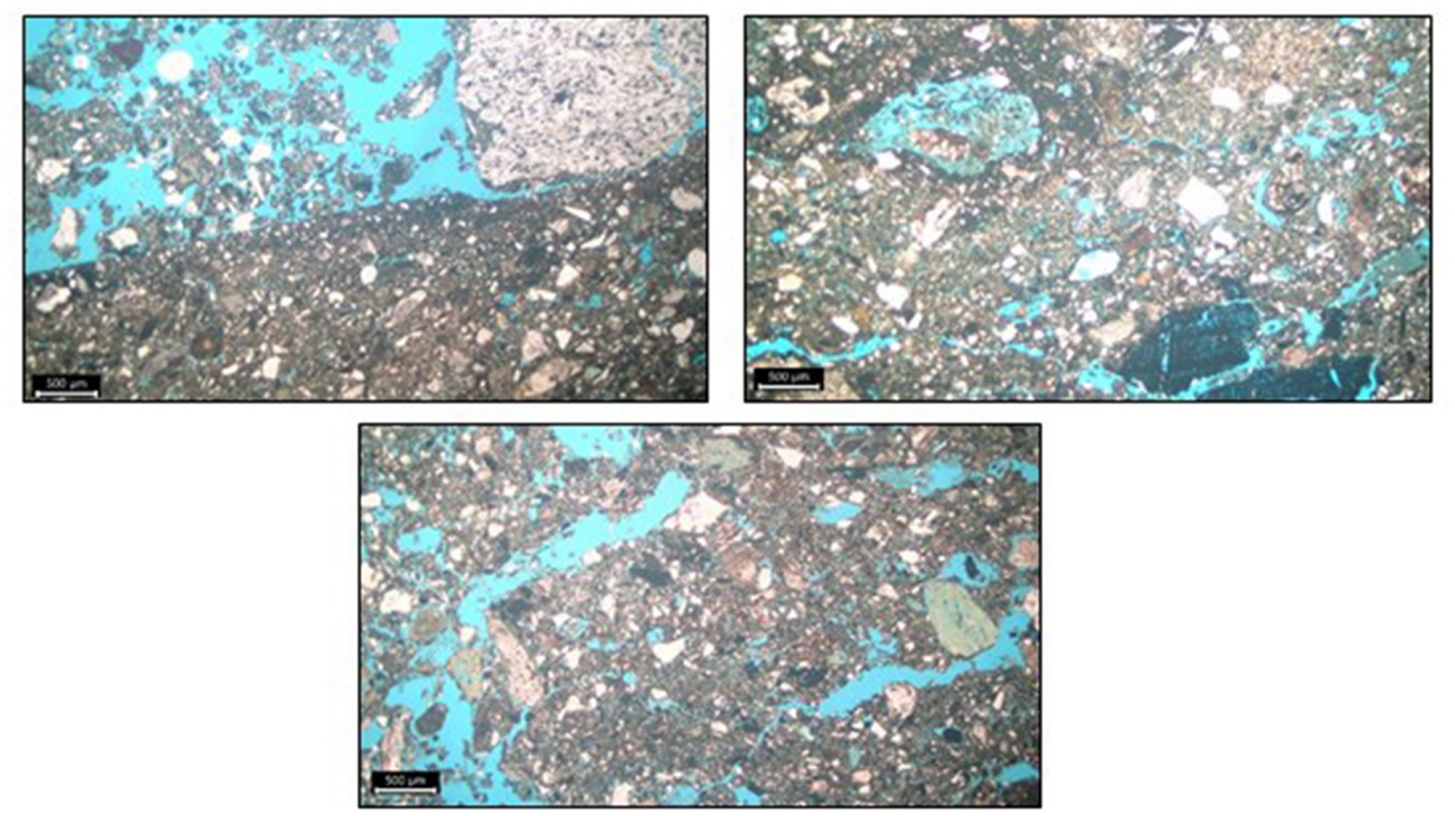
Micromorphology images showing bedding within Feature B14 (**top left**), Fe/Mn coating and hypocoating around a vugh void and burned plant material (**top right**), and bone (**bottom left**). All photographs are from Feature B14, taken at 2.5x magnification in plane polarized light.

Micromorphological studies were designed to provide insights into sediment content and structure not possible with simple non-microscopic studies. We chose an example of a bedded (B14) and non-bedded (C5) pit for micromorphological examination (Figure 5). The micromorphological samples from B14 show microscopic evidence of bedding visible on the macro scale. In general, samples from Feature B14 are matrix supported, with a massive microstructure; burned plant inclusions (Xylem) and bone, consistent with fishbone, are frequent in these samples. Though the sample has evidence for bedding, the matrix is relatively homogenized, indicating bioreworking of much of the sample. However, FE-MN coatings and hypocoatings are observed lower in the profile, providing evidence that post-depositional processes within this feature do remain at least partially intact. The FE-MN coatings and hypocoatings, with the absence of both nodules and depletion features, indicate inundation of the contents of the pit. This would suggest that the pit contents remained exposed for perhaps days at a time, though not for prolonged period (96). With significant organic content evident in these samples and greater concentrations of debris likely derived from the adjacent kitchen floor (burned plant debris and bone), use of the B14 pit was likely intensive.

The micromorphological samples from Feature C5 generally have less bone and burned plant material comprise less material within the sample. The open, disorganized structure of the sample is relatively homogenized above channel voids are most common throughout, indicating consistent bioreworking. The sample is more depleted of organic content and pedofeatures are less common. The micromorphological analysis of C5 suggests that it was less intensively utilized in comparison to B14.

Collectively, these results suggest two quite different fill scenarios. Feature B14 has extensive crushed bone and plant materials and indicators of periodic exposure. In contrast, C5 has limited organic content and general indicators of low-intensity use. We suggest that B14 sediments may have accumulated via use of swept/cleaned floor material (complete with fish bone and plant content) dumped periodically into the pit, where it was crushed by purposefully human pressure (stamping for example). This inference is in line with a model of cyclical topping up the pit with new sediments for a new period of use, perhaps related to storage. In contrast C5 sediments are relatively clean yet associated with relatively abundant cultural materials, especially faunal remains. We suggest that the pit fill here may have been derived from substrate (sediments below the cultural layers) and used to top-up and fill-in the C5 refuse pit. Thus, while we have only looked at two pit features through a micromorphological lens it does suggest that macroscopically bedded pits may have been filled under very different conditions than more homogeneous pits.

#### 4.1.3 . Pit type: sediment geochemistry

Stable carbon isotope (δ^13^C) values were broadly consistent across all pit features. Values ranged between −27‰ and −22.5‰, and showed no relationship with sample depth. In contrast, nitrogen isotope (δ^15^N) values exhibited substantially greater variability (6.1‰−19.1‰) and a consistent negative correlation with depth. Samples from pit features from deeper levels tended to have lower δ^15^N values than those near the surface. This pattern likely reflects the sequential accumulation of household refuse, where earlier (deeper) deposits represent less isotopically enriched material from earlier activity episodes.

Comparisons among pit features revealed significant differences in both δ^13^C and δ^15^N values. This was primarily driven by two bedded features (Features B14 and B15). These distinctions suggest variation in depositional or post-depositional processes between bedded and non-bedded features, potentially reflecting differences in refuse management, depositional timing, or organic input. Importantly, δ^13^C and δ^15^N values were not significantly correlated, consistent with the expectation that multiple food sources and mixed anthropogenic inputs contribute to isotopic variation in pit fill rather than the more predictable trophic relationships seen in faunal tissues.

Elemental analyses support these isotopic trends. Ratios of calcium (Ca/Ti), potassium (K/Ti), and phosphorus (P/Ti) were used to evaluate variation in midden and clean-up from hearth-related activity. Calcium concentrations generally increased with depth, while phosphorus decreased, suggesting differential leaching or changing inputs through successive depositional episodes. Potassium showed no clear depth-related pattern. Together, these results reinforce a pattern of stratified refuse accumulation, with geochemical variation reflecting both depositional sequence and activity type.

#### 4.1.4 . Pit type: summary

Considering results of pit bedding, sediment micromorphology, artifact and faunal contents, and geo-chemical and isotopic assessment, we conclude that it is likely that pits B14, B15, and A1 mostly formed through a different process than the others. Bedding, sediment content and structure, low artifact and faunal frequencies, and significant differences in δ13C and δ15N between B14 and B15 vs. the others implies that these pits may have accumulated through cycles of use as storage facilities leading to the establishment of beds resulting from re-starting the pit multiple times after previous use periods. This would have resulted in lowered contributions of macroscopic kitchen materials as compared to refuse disposal pits. However, though drawn from a limited sample, micromorphology assessment suggests that storage pits accumulated redeposited floor material while refuse pits were topped up with semi-sterile sediments perhaps to bury odorous kitchen refuse. Consequently, we drop B14 and B15 from the analyses of contents. Even though sediment samples were not available from A1, its sedimentary structure and generally low artifact and faunal elements scores suggest it formed in a similar manner to the former two pits.

### 4.2 . Pit contents

We infer variation in kitchen regimes from refuse pits as indicated by faunal and floral remains and geochemical signatures.

#### 4.2.1 . Pit contents: faunal remains

Given our desire to interpret kitchen regimes on floors, faunal data from accepted refuse pits were organized on a floor-by-floor basis meaning we combined data from features when derived from the same floor (original faunal data can be found in “Housepit 54 Project, Bridge River site, British Columbia” by Anna Marie Prentiss). The data (Table 2) were then subjected to a PCA yielding two significant components (Table 3). Because we produced two principal components (PC), we rotated the component matrix to enhance interpretation (Table 4) (97). Finally, we retained component scores to aid in interpreting data for each floor (Table 5).

**Table 2:** Data used in Principal Components Analysis (PCA).

**Feature(s)**	**Floor**	**Variable Number (see below)**							
		**1**	**2**	**3**	**4**	**5**	**6**	**7**	**8**
		**MC**	**MAPP**	**MAX**	**MD**	**FCr**	**FT**	**FCa**	**FD**
D8	IIc	0.03	0.76	0.21	0.14	0.40	0.23	0.33	0.84
D16	IId	0.01	0.99	0.00	0.71	0.50	0.18	0.26	0.97
D20, D11, B3	IIe	0.02	0.92	0.06	0.11	0.36	0.30	0.26	0.61
A17	IIf	0.14	0.43	0.43	0.02	0.13	0.59	0.00	0.06
A5, C10	IIh	0.07	0.64	0.30	0.10	0.40	0.06	0.16	0.62
C5	IIk	0.07	0.85	0.08	0.23	0.40	0.19	0.36	0.92
A11	IIl	0.04	0.73	0.07	0.36	0.53	0.14	0.22	0.44
A12, A14	IIm	0.20	0.67	0.13	0.31	0.37	0.21	0.38	1.26
A17	IIn	0.00	0.00	0.00	0.00	0.76	0.15	0.09	2.70

MC, Mammal Crania index; MAPP, Mammal Appendicular index; MAX, Mammal Axial index; MD, Mammal Density; FCr, Fish Crania index; FT, Fish Thoracic index; FCa, Fish Caudal index; FD, Fish Density.

**Table 3:** Basic PCA statistics.

**Component**	**Total**	**Eigenvalues**	**Cumulative**
		**as % of variance**	**%**
1	3.486	43.572	43.572
2	2.479	30.984	74.556
3	0.906	11.321	85.876
4	0.554	6.93	92.806
5	0.435	5.433	98.239
6	0.097	1.218	99.458
7	0.043	0.534	99.992
8	0.001	0.008	100.00

**Table 4:** Rotated component matrix associated with the PCA.

**Variable**	**Component**	
	**1**	**2**
1	0.634	−0.049
2	0.269	0.928
3	0.826	−0.403
4	−0.177	0.795
5	−0.984	−0.088
6	0.733	−0.344
7	−0.159	0.805
8	−0.832	−0.345

**Table 5:** Component scores from the PCA.

**Feature**	**Floor**	**PC1**	**PC2**
D8	IIc	0.12229	0.2234
D16	IId	−0.53484	1.30123
D20, D11, B3	IIe	0.17312	0.35993
A17	IIf	1.96961	−1.37468
A5, C10	IIh	0.21371	−0.31070
C5	IIk	−0.03965	0.66878
A11	IIl	−0.30547	0.45536
A12, A14	IIm	0.28944	0.48089
A17	IIn	−1.88821	−1.80421

On PC1 most significant components scores in the positive dimension load on artiodactyl crania and axial measures along with the fish (salmonid) thoracic index. In the negative dimension, strongest loadings occur on salmonid crania and overall density. On PC2, we recognize strongest positive scores on artiodactyl appendicular, artiodactyl density, and salmonid caudal indices. Strongest negative scores are present for artiodactyl axial and salmonid thoracic measures. Summarizing these results, we identify three major patterns in these assemblages. Type 1 assemblages include a strong artiodactyl cranial and axial signature coupled with salmonid thoracic sections. Type 2a assemblages are dominated by salmonids in general including cranial parts and have very little contribution from artiodactyls. Type 2b is also salmonid dominant with a strong contribution by caudal parts along with artiodactyl appendicular parts.

As summarized on Table 6, these results can be plotted against floors by use of component scores. Assemblage Type 1 occurs uniquely on floors IIf and IIh. Unfortunately, we were unable to assess floor IIg. Assemblage Type 2a is found exclusively on IIn. Finally, assemblage Type 2b is found on floors IIm to IIk and IIe to IIc. In general, these patterns suggest that long-distance hunting trips with extensive field processing coupled with intensive transport of salmonid parts that included lower utility elements (caudal) were the norm in the history of Housepit 54 kitchens given this pattern on six of the nine floors examined. Interesting exceptions are associated with Type 1 on IIh and IIf where artiodactyl axial/cranial and high utility salmonid parts are dominant; and Type 2a where salmonids are nearly totally dominant over artiodactyls.

**Table 6:** Faunal assemblage types in context.

**Floor/ feature context**	**Faunal**		
	**Assemblage type**	**General occupation period and estimated date** ^a^	**HP54 form**
IIc	2b	Mid-BR3/1171 cal. BP	Large oval
IId	2b	Mid-BR3/1195 cal. BP	Large oval
IIe	2b	Early BR3/1219 cal. BP	Large oval
IIf	1	Early BR3/1243 cal. BP	Rectangle
IIh	1	Early BR3/1292 cal. BP	Rectangle
IIk	2b	Late BR2/1364 cal. BP	Rectangle
IIl	2b	Late BR2/1388 cal. BP	Rectangle
IIm	2b	Mid-BR2/1412 cal. BP	Small oval
IIn	2a	Mid-BR2/1436 cal. BP	Small oval

^a^Estimated occupation date is based on data from Prentiss et al. (75).

#### 4.2.2 . Pit contents: botanical remains

Berry seed data are summarized on Tables 7, 8. As has been recognized previously in other Housepit 54 contexts (104) berry seeds occur relatively infrequently. Only pit features from three floors (IIe, IIf, and IIh) had enough seeds to draw any conclusions regarding patterns. All are dominated by *Arctostaphylos uva-ursi* (kinnikinick or bearberry) and very limited numbers of others including *Amelanchier alnifolia* (Saskatoon serviceberry), *Prunus* sp. (chokecherry, bitter cherry, or pin cherry), *Rubus* sp. (raspberry, blackcap, or thimbleberry), *Sambucus* sp. (elderberry), and *Viburnum* sp. (cranberry). These data suggest that during the early BR3 focus on local artiodactyl predation (faunal assemblage Type 1 on IIh and IIf), groups also routinely harvested local berries. Berry taxa designated as famine food concentrate on IIm and IIf. Their presence on IIm aligns with both climate and population expectations. The high count on IIf is not a good fit for either and suggests that we have much more to learn about inter-annual variation that is difficult to monitor even in the contexts of the generational floors at Housepit 54. We must also recognize however that given wide differences in representation of seeds in pits, there could be significant variation in refuse disposal practices with reference to plant remains.

**Table 7:** Berry data.

**Feature**	**Floor**	**Am**	**Arc**	**Eric**	**Prunus**	**Rosa**	**Rub**	**Sam**	**Sorb**	**Vib**
B3, D20	IIe		52							
A17	IIf	1	13		1					
A5, C10	IIh	3	24	1			1	1		0.5
C5	IIk		1					0.5		
A14	IIm		1			1			1	

Am, *Amelanchier* sp. (Saskatoon, edible); Arc, *Arctostaphylos* sp. (kinnikinick, edible); Eric, *Ericaceae* sp. (heather, medicinal); *Rosa, Rosaceae* sp. (rose, medicinal); Rub, *Rubus* sp. (raspberry, edible), Sam, *Sambucus* sp. (elderberry, edible); Sorb, *Sorbus* sp. (mountain ash, edible in limited quantities); Vib, *Viburnum* sp. (cranberry, edible).

Plant utility distinctions from Turner (98–100), Turner and Bell (101), Turner and Davis (102), and Turner et al. (103).

**Table 8:** Plant utility data (combines all plant data including berries) by floor.

**Floor**	**Edible**	**Medicinal**	**Technological**	**Famine**	**Multipurpose**
IIe				6	62
IIf	3		6	20	13
IIh	5.5	1	2	7	24
IIk	0.5				1
IIm	7	1	3	12	0.5
All	16	2	11	45	100.5

#### 4.2.3 . Pit contents: sediment geochemistry

To explore potential relationships between pit-fill isotopic composition and variation in food consumption or refuse disposal practices, we applied a SIMMR isotope mixing model (105, 106). Full analytical parameters, input datasets, and model diagnostics are presented in Supplementary Materials A. Food sources were grouped into three composite categories—domestic dog, terrestrial mammals (mule and white-tailed deer, bighorn sheep, beaver, elk, moose), and salmon to capture the dominant isotopic contrasts observed in the faunal assemblage.

The model exhibited a poor overall fit (see Supplementary Materials A, Supplementary Figure S3), likely due to limited isotopic separation among source groups (particularly the overlap between domestic dog and salmon) and because SIMMR was designed for tissue-based data rather than sediment matrices influenced by anthropogenic activity. Additional uncertainty arises from the absence of local plant isotope baselines and the mixed nature of domestic sediment inputs.

Despite these constraints, several patterns are apparent. δ^13^C values were relatively consistent among pit features, while δ^15^N values showed greater variability and a significant relationship with depth: deeper samples generally exhibited lower δ^15^N values (see Supplementary Materials A). This trend suggests that early pit-fill deposits represent earlier floor-cleaning or disposal events with less cumulative isotopic enrichment. Comparisons among pits indicate marginal differences in δ^15^N values—most evident in the deepest levels—supporting the interpretation that isotopic variation reflects temporal differences in household activity rather than differences in species composition alone.

At the feature scale, average δ^13^C values correlated positively with faunal density (rs = 0.685, *p* < 0.05), while δ^15^N values did not (Supplementary Materials A). This pattern implies that carbon isotopic variation may partially reflect the overall magnitude of food refuse deposition. In contrast, element ratios (Ca/Ti, K/Ti, P/Ti) showed no significant relationship with faunal density, indicating that elemental enrichment likely integrates multiple activity sources and post-depositional processes rather than direct food waste inputs.

Phosphorus concentrations decreased with depth; a pattern consistent across features (Supplementary Materials A). This gradient may reflect sequential cleaning and infilling of domestic floors—deeper, earlier deposits capturing less enriched sediments and upper levels reflecting cumulative activity or potential re-use of filled pits as active kitchen surfaces.

Overall, isotopic and elemental variation in pit sediments captures subtle differences in the tempo and character of domestic activity, especially food preparation, disposal, and floor maintenance that are not readily visible through faunal data alone. The δ^15^N values, in particular, highlight distinctions among depositional histories and subsistence behaviors, underscoring the interpretive value of sediment geochemistry for reconstructing household practices.

In reference to hypothesis expectations, if we exclude surface/shallow pit data that is probably affected by random kitchen activities, we actually recognize some alignment with the faunal assemblage outcomes. Thus, δ^15^N values implicate similar fish (salmonid) contributions across the IIn-IIk and IIe-IIa floors. For the IIh floor, where pit fill is predominantly transported axial and cranial sections from artiodactyls, δ15N values are fairly similar to those found for the fish dominated pits. However, this may be due to the utilization of canids as an additional foodstuff whose isotopic signature would have been closer to fish than that of other mammals given that domesticated dogs were often fed salmon. This aligns with the fact that feature A5 has the highest count of identifiable canid remains from the study pits. The other Type 1 assemblage pit of Feature A17 for floor IIf has high δ15N values, much lower δ13C values, and is most similar to the B3 pit from the IIe floor which is an isotopic outlier based on statistical analysis. Since B3 is one of three pits from the same floor, it's distinctive isotopic signatures may reflect floor activity or cleaning activity variance.

#### 4.2.4 . Pit contents summary

Analyses of pit stratigraphy and sediments, artifact and faunal contents, and sediment geo-chemistry permitted us to identify 13 of 16 pits as associated with refuse disposal. The three other pits are likely long-term use features for food-caching. Data from micromorphology and sediment geo-chemistry and isotopic analyses implicate variability in the 13 refuse pits associated with kitchen activity and associated cleanup. Analyses of artiodactyl and salmonid elements confirms three distinct assemblage types: artiodactyl axial part and salmonid thoracic part (1), salmonid dominated with little artiodactyl (2a), and salmon with artiodactyl appendicular parts (2b). These results are generally replicated by isotopic signatures from deeper strata in pits as upper strata are likely confounded by a random assortment of kitchen factors. Element concentrations are not distinguishable between different faunal assemblage groups showing consistency in general household activities and kitchen cooking strategies/methods. Botanical remains suggest a consistent lower elevation valley focus on berry harvest during the time of particularly focused local artiodactyl hunting and for one generation thereafter (IIh-IIe).

## 5 Discussion

Materials extracted from the deep pits in Housepit 54 permitted analyses of general pit contents, sediment structure, and geochemical signatures. We now focus on their implications for explanatory scenarios and general issues associated with interpretation of deep pits from Mid-Fraser area housepits.

**Figure 6:**
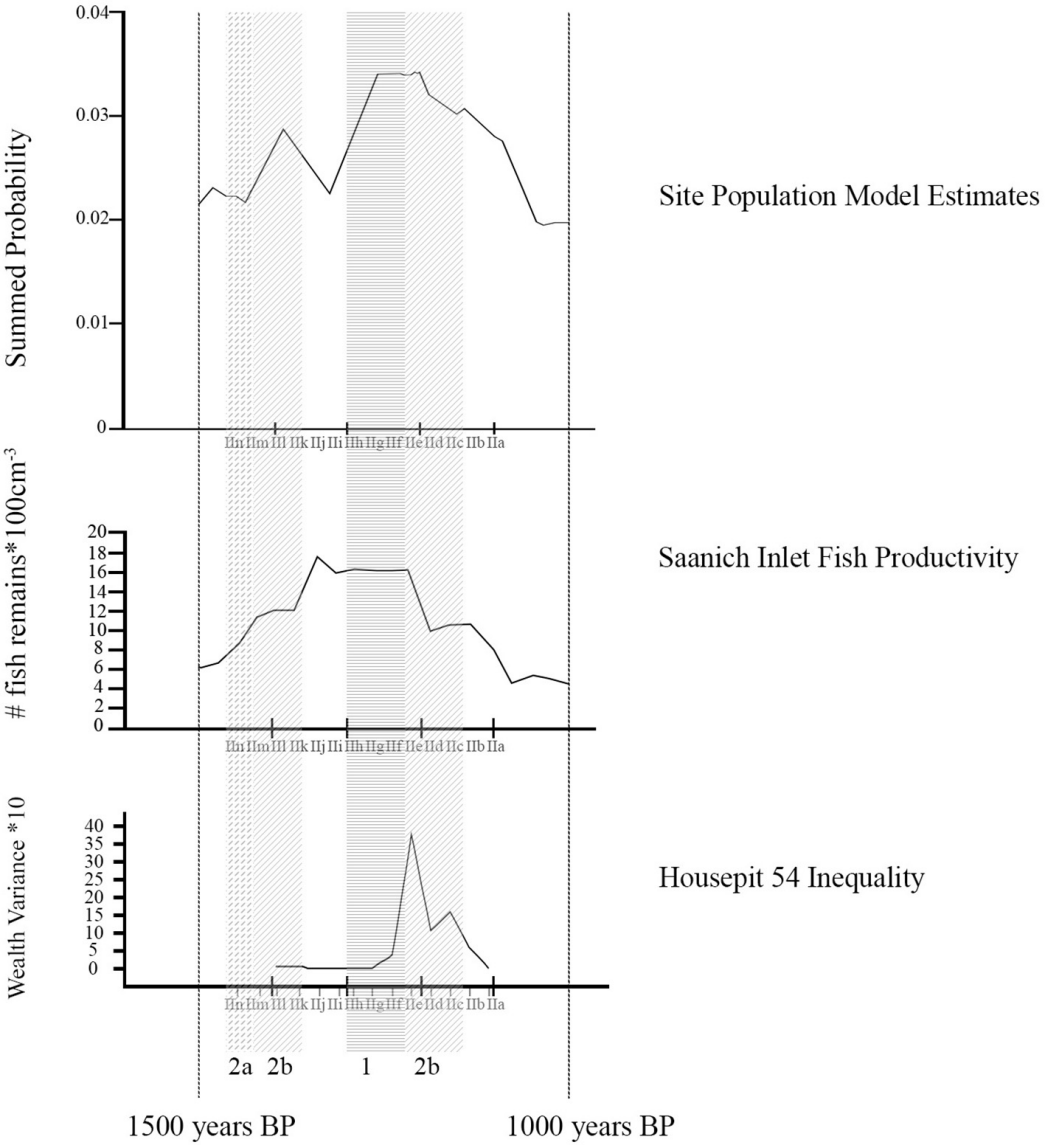
Faunal assemblage types associated with Housepit 54 floors plotted against population, climate, and social inequality models (see Figure 3). Faunal assemblage Type 1 refers to an axial dominated signature for artiodactyls and thoracic signature for salmonids. Type 2a refers to nearly total salmon dominance. Type 2b identifies appendicular artiodactyl signature and full salmonid (caudal parts are particularly strong) pattern. Type 1 faunal assemblages are also associated with valley bottom berry profiles. Type 2a and 2b faunal assemblages occur in pit sediments with isotopic signatures of strong fish contributions.

As illustrated in Figure 6, we see that faunal assemblage Type 1 with its emphasis on transport of axial and cranial sections from artiodactyls along with thoracic sections of salmonids falls in the early BR3 period (IIh-IIf) as populations are rebounding after the late BR2 low. Assemblage type 2b with its emphasis on appendicular parts and all salmonid (especially caudal) elements, dominates most of the rest of the assemblages thus associated with either high (mid-BR2 and BR3) or declining (late BR2 and mid-BR3) human populations. The one Type 2a representative on IIn is anomalous and either represents sampling error or a single generation benefitting from exceptionally strong salmon returns. Setting the latter aside, the pattern replicates the predictions of the population model, that optimal local artiodactyl hunting would occur after a human population low (reduced pressure on local game) and that in turn this would shift priorities with regards to transport of salmonid elements. The climate model is not entirely rejected as clearly salmon fishing was strong during early BR3 growth (IIh-IIf) period aligning with likely strong marine fish production. However, climate impacts on potential food resources were mediated by local population dynamics and associated pressure on terrestrial prey populations followed by trade-offs on approaches to acquisition, processing, and transport of fish resources. The social hypothesis is not strongly supported as concentrations of highest utility artiodactyl (entire carcass representation) and salmonid (thoracic concentrations) remains occur on the floors pre-dating the appearance of material wealth-based inequality and its effects on household cooperation and coercion strategies.

While it is well known that St'át'imc fishermen and women had a variety of procedures they could choose from to process salmon caught at riverside fishing sites (46), no one has assessed whether or not there was variability in the elements (and thus sections of each fish) transported and why that might be. Our study suggests that decisions favoring one section (cranial, thoracic, caudal) or another for transport was conditioned not only by the overall utility of each part (combined meat and fat value) (75, 84), but also those factors in light of decisions with regard to accessibility and treatment of other food resources. Thus, in this case, we infer that during the occupation period associated with the IIh to IIf floors (also known as the early BR3 period at the Bridge River site), local artiodactyl populations appear to have rebounded allowing hunters to be highly productive without long trips or extensive field butchery. Given the glut of comparatively easily obtained mammal products, fish processors often chose to transport to the house only the highest utility fish segments (thoracic) due to their high fat content and thus, good flavor. Then, when hunting conditions were more challenging (mid-BR2 and mid-late BR3) hunters had to travel further afield requiring more intensive field butchery and more frequent transport of appendicular parts. Because of the extra effort required in obtaining game meat, it then made more sense for fish processors to bring back more whole (read thoracic and caudal) parts of fish. The reader should be aware however, that these transport decisions really focused on backbone sections or as they are often termed today, “neckties,” such that while all fish meat would be dried and transported as *tswan*, backbone sections were primarily transported for lean season backup, special soups, or dog food (31). Thus, the decision to primarily transport thoracic backbone sections really meant that times were especially good and there was no anticipated lean season. Rather, there was an expectation that fatty backbone sections could be used in much-desired soups.

Local valley-bottom berry resources were harvested most intensely during the time when we infer local artiodactyl hunting was at its best (IIh-IIf) with continuation of the same pattern into floor IIe. This aligns with Prentiss et al. (74), who recognized a pattern of change in the berry record in a nearby village (Tl'atl'lh [Keatley Creek]), whereby local mammal and fish predation activities favored harvest of local berries and more diverse hunting strategies also encouraged greater harvest of more distant berry species. Presence of famine associated taxa (Tables 6, 7) on select floors is provocative but at this point inconclusive in reference to broader hypotheses.

Returning to the explanatory scenarios, it is evident that the material conditions of food procurement played a greater role in foraging, fishing and associated processing/transport decisions and behaviors than did social change we recognize beginning on the IIe floor of Housepit 54 (87). This does not negate the processes by which material wealth-based inequality developed in Housepit 54 or across the K'etxelknáz (Bridge River) village (107, 108). Indeed, these results actually shed additional light on that process. Prentiss et al. (75) argue that wealth inequality emerged during a second Malthusian ceiling during which village population had peaked and then begun to decline due to instability in the salmon fishery and costs for gaining access to other resources, especially artiodactyls. Inequality was initially cooperative in nature linked to house level alliances between families. This shifted quickly to a more coercive model under conditions of rising resource cost and the possibility that select social groups may have controlled access to better procurement locations (fishing, hunting, and gathering sites) (90). Data developed in this study support the cyclic model recognizing that the first Malthusian ceiling was resolved by greater winter residential mobility (75) thus providing the opportunity for local game populations to return and rebound, in turn setting the stage for the early BR3 demographic growth phase under conditions of subsistence activity leading to what we here call Type 1 faunal assemblages. While the current study does not address the ca. 1,100 cal BP abandonment of Housepit 54 and subsequent depopulation of the overall village it does suggest predation and processing decisions at that time were in line with previous high population density cycles. In both scenarios, families ultimately decided to leave the aggregated village most fundamentally to maintain adequate nutrition and returned only when conditions were more favorable. At the end of the BR2 cycle the village was never fully abandoned while at the end of BR3 the occupation of the village appears to have been exceptionally sparse (89) such that while it likely remained a significant part of the cultural landscape, very few people chose to live there. Put differently, the early BR 3 (Housepit 54 IIh-IIf) period represents a subsistence anomaly that favored a particularly well-fed and growing population before the demographic peak and turn on floor IIe at just over 1,200 years ago,

We also consider the archaeology of deep pits on house floors. These features are generally assumed to be storage features or “cache pits” whether in the archaeological (6, 8, 32, 82) or ethnographic (33) records. Our results suggest that pits had diverse fill histories. These range from scraping up kitchen floor material to partially filling and re-setting a pit for a new cycle of storage to dumping fire-cracked rock, lithic tools, and faunal/floral remains. In some cases, this could include dumping of relatively sterile fill, perhaps to eliminate odor. This suggests not all deep and wide pits may been storage features. At Housepit 54 we only recognize three features mostly recycled as multi-use cache pits. Two are on floor IIe and the other on IIg. The particularly voluminous IIe pits (B14 especially) imply regular movement of abundant food into the house, which makes sense given peak population is projected for that floor. If in-house storage was otherwise relatively rare, then this offers implications for the demand and availability of winter foods. It may be that the norm was to store foods outside in the village and in procurement locations and move those foodstuffs into the house only when needed for direct consumption. Thus, variation in the locations of voluminous pits might not be reliable indicators of differential wealth as suggested elsewhere (6). This topic requires substantial further research as cache pits on IIe could have also been associated with emergent wealth differences and an associated feasting complex. Minimally, we can no longer assume that deep pits always implicate food storage. Some or even many may be waste receptacles.

Finally, placing this study in its wider regional context, we recognize that reliance upon salmon harvest for storage and winter survival was ubiquitous across the Pacific Northwest. Suttles (109) recognized the importance of storage preparation with regards to salmon flesh, oil, and eggs for Coast Salish groups. Teit (40, 41) described the critical practices of salmon fishing and drying for winter consumption in the Mid-Fraser area. Kennedy and Bouchard (46) and Romanoff (44) outline in detail diverse fishing and storage traditions of the St'át'imc people. Archaeological evidence for intensive fishing and mass storage of salmonids on the Coast and Interior is widespread [e.g., (48, 76, 110–112)]. Yet, to our knowledge, no archaeological or ethnographic project has identified variability in the effects of field processing and transport of salmon as broken down into anatomical units (cranial, thoracic, caudal). Here [see also (75)], we recognize, that under times of general abundance of terrestrial and anadromous food resources, fishing people could afford to be selective in anatomical parts returned to the village. As noted by Prentiss et al. (75, 84), thoracic vertebral sections are particularly high in fat content making them most nutritious and flavorful as for example, when used in soups. Clearly, there was a cost-benefit factor associated with transport decisions (72) given the practice of selective transport of thoracic vertebral sections only occurred when hunted resources were very abundant. However, we raise the possibility that there was simultaneously a cultural desire for tasty food supplements [i.e., (42)] during a time of lower food risk and population growth. We encourage zooarchaeologists working elsewhere in and beyond the Pacific Northwest region to take a closer look at their assemblages of fish remains as there is more to learn.

## Data Availability

The datasets presented in this study can be found in online repositories. The names of the repository/repositories and accession number(s) can be found below: “Housepit 54 Project, Bridge River site, British Columbia” by Anna Marie Prentiss, https://scholarworks.umt.edu/household_archaeology_supplemental_data/.
