# Supplementary material for: Dietary change revealed in kitchen refuse pits from the ancient floors of Housepit 54, K'etxelknáz (Bridge River Site), British Columbia
Source: Front Nutr. 2026 Jan 29;12:1716684. doi: 10.3389/fnut.2025.1716684 (PMC12926657; doi:10.3389/fnut.2025.1716684)
Supplement: Supplementary file 1 [file Presentation_1.pdf]

Supplementary Materials A

Methods for Collecting and Processing Sediment Samples

A minimum of 1 sample was taken per pit feature. Additional samples were taken if the feature expanded across multiple quads so that a sample from each quad was also included. Usually at least 1 sample per every arbitrary level was taken as well (Table 1).

Pit Feature	Excavation Year	Floor	Total Number of Samples Included in Analysis
D8	2014	IIc	9
D16	2016	IId	9
D20	2016	IIe	14
D11	2016	IIe	9
B14	2014	IIe	11
B15	2014	IIe	6
B3	2014	IIe	3
A17	2013	IIf	1
A5	2014	IIh	5
C10	2022	IIh	4
C5	2016	IIk	4
A11	2016	IIL	4
A14	2016	IIm	5
A12	2016	IIm	4
A17	2016	IIn	2

Table 1 List of Feature Samples Included in Analysis

Each sediment sample of approximately 250g was first sifted through a 4 mm sieve to separate out larger grains and any faunal/lithic/non-sediment material that may have been included in the sample. The smaller grains were then packaged for further processing for both isotope and x-ray fluorescence analysis.

<u>Preparing Sediment Samples for Isotope Analysis</u>

For each sample, 10mL of sediment were prepared for Isotope Ratio Mass Spectrometry using a standard acid wash to remove organic carbon prior to analysis. 10mL of HCL and 20mL reverse osmosis water was added to each 10mL sample. The samples then sat for 24 hours. After the 24 hours, the liquid was decanted from each sample, and 30mL of reverse osmosis water was added. This process was repeated six times. After six cycles, the pH of the samples was taken, and if the pH was neutral, the samples were ready for analysis. If the samples were still acidic, the decanting and addition of reverse osmosis water cycle repeated until the pH was measured to be neutral. The samples were then placed into a 50 degree Celsius oven for 24 hours to remove any reverse osmosis water remaining. Each sample along with laboratory standards was then weighted in accordance to carbon and nitrogen ratios on a Sartorius micro-balance, and placed in aluminum cups. The samples were then loaded into the Elemental Analyzer autosampler for IRMS analysis. Analysis was performed using Thermo Scientific Delta V Advantage Isotope

Ratio Mass Spectrometer. Standards were calibrated against NIST Standard Reference Materials. Preliminary values for each sample were normalized and reported according to the International Stable Isotope Reference Scale, based on the known value of laboratory standards.

Preparing Sediment Sample for XRF Analysis

To prepare the samples for XRF analysis, the sediments had to be heated to eliminate any remaining organic carbon. Each sample was placed in an aluminum tray wiped with ethanol to prevent contamination. The samples were then placed in a 50 degree Celsius oven for a week, then repackaged in clean plastic cups. The sediment was ground in a RockLabs tungsten carbide ring mill for a duration between 30 seconds and 2 minutes, based on the weight of sample, and packaged in clean plastic cups and labeled.

For analysis, 5 grams of sediment were placed in SPEX CertiPrep 31mm X-Cell plastic cups, ensuring the sample was not compressed. The cups were then covered with 4 micron thick ULTRALENE clear film. The film was secured with a plastic snap-on ring. Data for concentrations of the following elements were recorded for each sample: Na2O, MgO, Al2O3, SiO2, P2O5, S, K2O, CaO, TiO2, MnO, Fe2O3, Cu, Zn, Br, Rb, Sr, Zr, Ag, and Pb. Samples were analyzed using Thermo ARL Perform'X EDXRF spectrometer at Hamilton College. The in-house EDXRF instrument was calibrated for archaeological research. It was calibrated using seven international standards including National Institute of Standards and Technology (NIST 278), the United States Geological Survey (RGM-1), the University of Georgia Center for Applied Isotope Studies (GBOR-01, MTNM-01, SATU-07) and an in-house standard (OBS-1 Glass Butte, OR).

Methods for Analyzing Sediment Sample Results

Results from Isotope Ratio Mass Spectrometry provided δ13C (‰) and δ15N (‰) isotope values per sample. These isotope results for each pit feature are in **Dataset 1-Isotope Results.** Selected element composition for each pit feature are in **Dataset 2-Element Results**.

The pit feature isotope values were compared using both one-way MANOVA tests with Tukey's HSD post-hoc test and non-parametric Kruskal-Wallis ANOVA followed by Bonferroni adjustment to test for pairwise differences between features. Statistical analysis was performed using SPSS (Version 29). Level of significance was set at $p \leq 0.05$.

The pit feature major and trace element values were compared using a non-parametric Kruskal-Wallis ANOVA followed by Bonferroni adjustment to test for differences between features based on measures of 3 elements (Ca, K, and P). Statistical analysis was performed using SPSS (Version 29). Level of significance was set at $p \leq 0.05$.

Results: Comparing Pit Features (Isotopes)

Boxplot distributions of the values for both δ13C and δ15N per feature indicate quite a few outliers, though the majority of features have a somewhat tight range with the major exception of Feature B14's δ15N values and Feature B3's δ13C values. All features have fairly consistent

values of δ13C with a constrained range between -27‰ to -22.5‰. Comparatively, there is more variation in δ15N values ranging between 6.1‰ to 19.1‰.

Isotope values were visualized by creating isospace plots of both δ13C and δ15N values per sample to examine for any clustering of values or outliers based on feature or floor (Figures 1-2). Overall the majority of samples at both the feature and floor level group together consistently based on their isotope values. Two of three samples from Feature B3, two Feature B15 samples, one sample from Feature B14, and the A17 (2013) sample do not cluster with the rest of the samples. Conversely, a majority of the samples from Feature B14 represent much lower δ15N (<12.0) but have similar δ13C values to the rest of the samples (between -23.0 to -24.0).

This clustering holds true when examining the values by floor, with these select features from Floor IIe occurring beyond the ranges of the other features. Feature A17 (2013) as the only pit feature analyzed from floor IIf similarly does not cluster with the rest of the features (Figure 2).

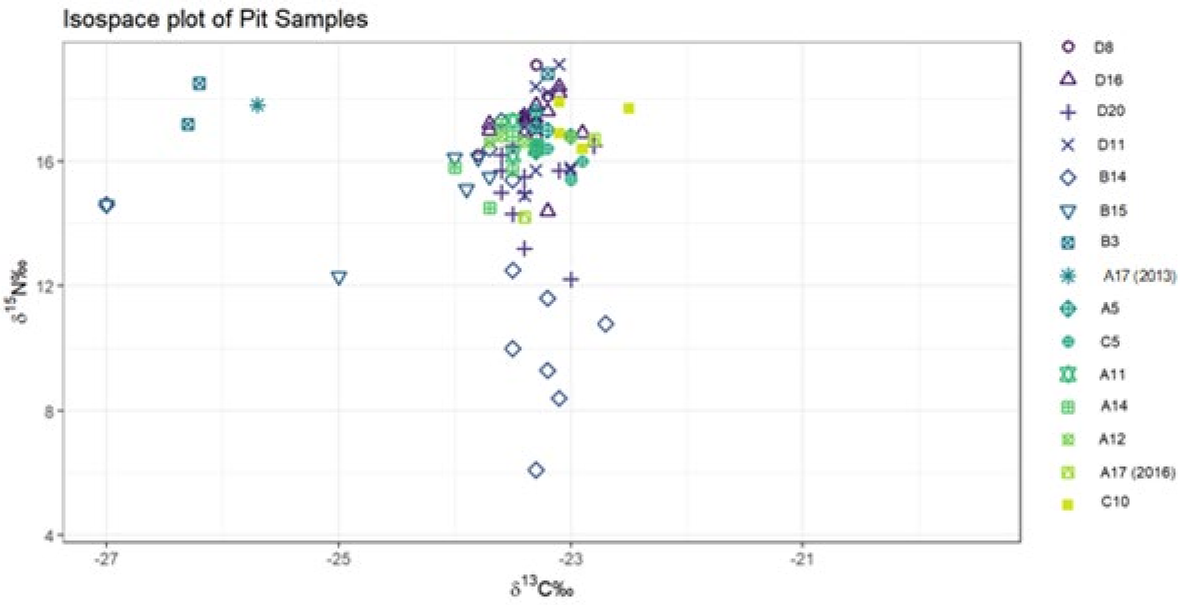

Figure 1. Isospace plot of pit feature samples by feature

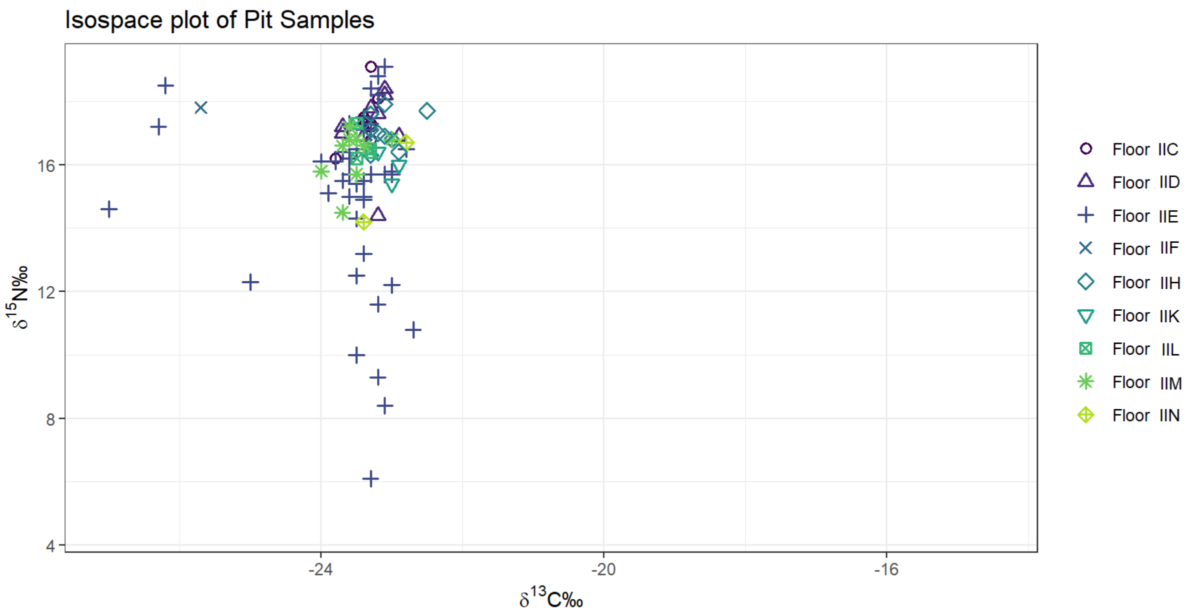

Figure 2. Isospace plot of pit feature samples by floor

For statistical comparisons, the singular sample for A17 (2013) was not included in the following tests. To compare distributions of individual isotope values across pit features, one-way ANOVA tests were performed and showed δ13C and δ15N values as being significantly different. **Results for** δ15N values was F(13, 75) = 6.703, p = <0.001 and for δ13C values was F(13, 75) = 3.958, p = <0.001.

Next, the non-parametric Kruskal-Wallis ANOVA results for δ13C (Kruskal–Wallis ANOVA, χ2 = 38.929, df = 13, P < .001) and δ15N values (Kruskal–Wallis ANOVA, χ2 = 44.476, df = 13, P < .001) indicate statistically significant differences as well. Pairwise comparisons of features with the Bonferroni adjustment shows which feature pairs have statistically significant differences. For δ15N values, Feature B14 was statistically significant from Features B3, D16 and D8 (Figure 3). For δ13C values, Feature B15 was statistically significant from Features C10, D11, and C5 (Figure 4).

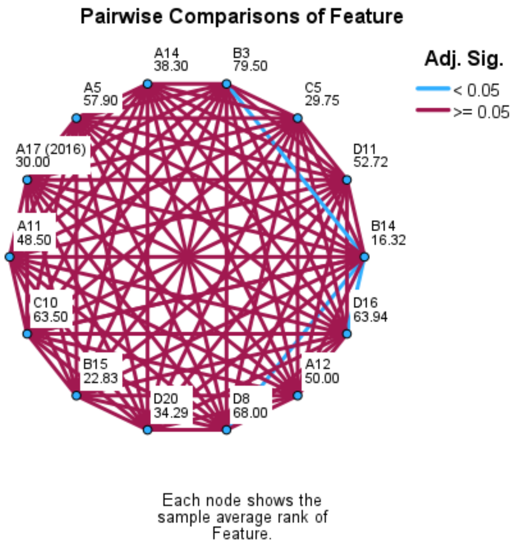

Figure 3. Kruskal-Wallis Test Pairwise Comparison for δ15N values with Bonferroni adjustment

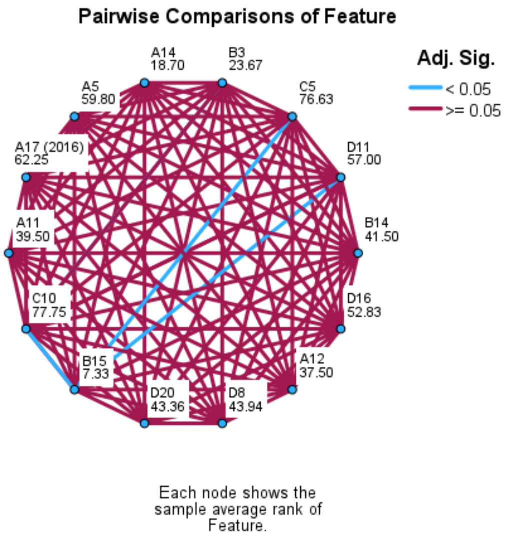

Figure 4. Kruskal-Wallis Test Pairwise Comparison for δ13C values with Bonferroni adjustment

To further compare values across pit features for δ13C and δ15N, a one-way multivariate analysis of variance (MANOVA) was performed. Results for both isotopes across features showed statistically significant differences, $F(26, 148) = 5.216$, $p < .001$; Wilk's $\Lambda = 0.272$, partial $\eta 2 = 0.478$. When using Tukey posthoc pairwise comparisons, this showed that for δ13C, Feature B15 was different from Features D8, D16, D20, D11, A5, C5 and C10 while Feature B3 differed from all other features except Features B15 and A14. For δ15N, Feature B14 was different from all other features except Features B15 and A17 (2016).

To compare isotope values to sample depth, Kendall's rank correlation tau was run in R (the cor.test function, method = kendall). This included using Dataset 1 both with Feature B14 and Feature B15 data included and running it without those two features as well considering their bedded nature may influence the isotope values per level. The δ13C values were found to be uncorrelated to sample depth while the δ15N variation appears negatively correlated to depth for all features, both when Feature B14 and B15 are included (τ_b = -0.325, p = 0.00003) and when the two features are not included (τ_b = -0.235, p = 0.007). Samples taken at deeper levels in a pit feature are more likely to have lower δ15N values than samples taken closer to the top of the pit.

Outliers Removed

The one-way multivariate analysis of variance (MANOVA) was run again with outlier values removed to help determine which feature(s) were statistically significantly different from each other and ensure statistical significance was not due to the influence of outlier values. This dataset is **Dataset 3-Isotope Results Outliers Removed**. The MANOVA test still indicated statistically significant differences, F(26, 128) = 6.792; Wilk's Λ = 0.177, partial η2 = 0.580. Tukey's posthoc analysis revealed statistically significant differences between Feature B3 and all other pit features for δ13C values and between Feature B14 and all other pit features for δ15N.

While the Feature B3 values may be due to the smaller sample size for that feature (n=3), other features with similarly smaller sample sizes did not show statistically significant differences. Importantly, for both the Kruskal-Wallis and MANOVA tests, Feature B14's δ15N values appear to be distinctive.

Lastly, to see if the δ13C and δ15N values are correlated, a Spearman's r correlation test was run. Results indicate that the isotopes are not statistically significantly correlated (*r_s = 0.113, p = 0.288).* Since multiple species/food contributions/anthropogenic and non-anthropogenic processes would be contributing to the isotopic values found in each feature fill, the typical relational connections between isotopes observed when studying measures from species (e.g. bone, collagen, etc) is not present.

Results: Comparing Pit Features Based on Faunal Assemblage Types (Isotope Data)

Statistical tests were also run to compare across the faunal assemblage groups with expectations that the two primarily fish groups (Type 2a and Type 2b) would be more similar while the primarily mammal group (Type 1) would evince statistically significant differences. Comparisons between pits showed no correlation between δ13C averages and faunal assemblage type while differences in δ15N averages were almost statistically significant (Kruskal-Wallis ANOVA χ2 = 5.0586, df =2, p-value = 0.07972). However, when we examine deepest samples per pit, the δ15N values were significantly different (Kruskal-Wallis ANOVA χ2 = 7.6154, df =2, p-value = 0.0222) across the faunal assemblage type groups while the top-level samples were not significantly different.

If pit fill is primarily from the same kinds of sweeping and depositing processes, then it may be that isotopic signatures are correlated with general faunal density regardless of faunal assemblage types. Utilizing the average value for each pit, Spearman's rho was calculated using

R (the cor.test function, method = spearman) to explore correlations between isotopes and faunal density. The average δ13C isotope values were found to be correlated to faunal density (r_s = 0.685, $p < 0.05$) while δ15N averages were not statistically significantly correlated to faunal density measures. Additional correlation tests included comparing the top level values per pit to faunal density and bottom level values per pit to faunal density. Where multiple samples were taken from a level, the values were averaged (Table 2). These comparisons provided similar results to the overall isotope average comparisons, with only top level δ13C isotope values correlating to faunal density (r_s = 0.640, $p < 0.05$). This shows that isotope signatures are related to subsistence variation, particularly δ15N values, and thus are reflective of food consumption fluctuations along with floor activity patterns, not just variance in the density of faunal material found in pit fill.

Feature	Top Level δ13C	Top Level δ15N	Bottom Level δ13C	Bottom Level δ15N	δ13C Average	δ15N Average	Faunal Assemblage Type	Faunal Density
D8	-23.4	17.4	-23.80	16.20	-23.377	17.433	2b	0.98
D16	-23.3	17.8	-23.20	14.40	-23.288	17.155	2b	1.7
D11	-23.2	18.56	-23.36	16.53	-23.233	16.855	2b	0.53
D20	-23.4	13.20	-23.50	14.30	-23.350	15.450	2b	1.54
B3	-24.7	18.65	-26.30	17.20	-25.233	18.166	2b	0.32
B15	-24.0	16.10	-23.80	16.10	-24.566	14.950	2b	0.005
B14	-23.7	16.40	-27.00	14.60	-23.663	12.036	2b	0.27
C5	-23.0	15.40	-23.10	15.90	-23.025	15.800	2b	1.15
C10	-23.1	16.90	-22.50	17.70	-22.900	17.225	1	2.58
A5	-23.2	17.00	-23.30	17.60	-23.220	16.960	1	0.86
A11	-23.4	16.85	-23.30	16.50	-23.400	16.600	2b	0.79
A12	-23.3	16.80	-23.10	16.60	-23.425	16.700	2b	0.66
A14	-23.6	17.20	-24.00	15.80	-23.660	16.000	2b	2.63
A17 (2013)	-25.7	17.80	-25.70	17.80	-25.700	17.800	1	0.07
A17 (2016)	-22.8	16.70	-23.40	14.20	-23.100	15.450	2a	2.69

Table 2. Summary Isotope and Faunal Density Data for Comparing Between Faunal Assemblage Types

Results: SIMMR Mixing Model (Isotope Data)

To explore the relationship between the δ13C and δ15N values for the pit feature fill and the possible faunal foodstuffs that would contribute to these values, a comparative dataset of known δ13C and δ15N isotope values for animals in the region based on both archaeological and modern samples was created using published data from Tifental 2016 and Schwarcz et al 2014 **(Dataset 4-Comparative Isotopes).** For values from Schwarcz et al 2014, only samples taken from species noted as being located in the Southern Interior were utilized to align as closely as possible with the location of the Bridge River site. SIMMR is a package for R which is an

upgrade on the SIAR package and is an isotope mixing model that attempts to estimate the proportion of different food sources contributing to a sample. In this case, the model was used to estimate the proportion of potential foodstuffs contributing to each feature fill (mixture) based on input isotopic values of δ13C and δ15N from these known foodstuff categories from this comparative isotope dataset. The source inputs the model compares to mixture samples are the mean and standard deviation of isotopic values of δ13C and δ15N for each food source (**Dataset 5-Isotope Food Source Averages and Standard Deviations**).

The comparative faunal isotope data was first mapped according to species, highlighting the isotope distinction between terrestrial mammals and fish, as well as how domestic dog values are most similar to salmon (Figure 5).

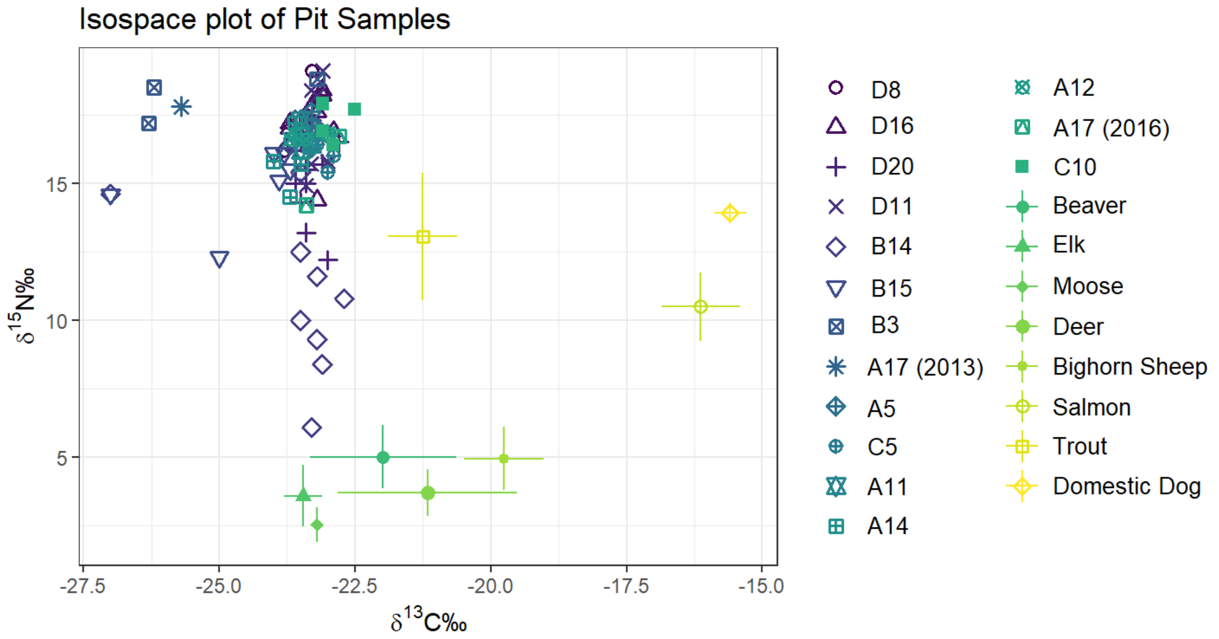

Figure 5. Isospace plot of pit feature values and faunal values

Next, foodstuffs were grouped into three primary categories of Domestic Dog, Terrestrial Mammals (mule deer, deer, bighorn sheep, beaver, elk moose), and Salmon to compress variation between food sources which would potentially work better for the SIMMR model and align with the overall variation expected between Fish/Terrestrial Mammal faunal remains observed in the pit fill. An isospace plot of these data along with all pit feature samples was also created for visual comparison (Figure 6).

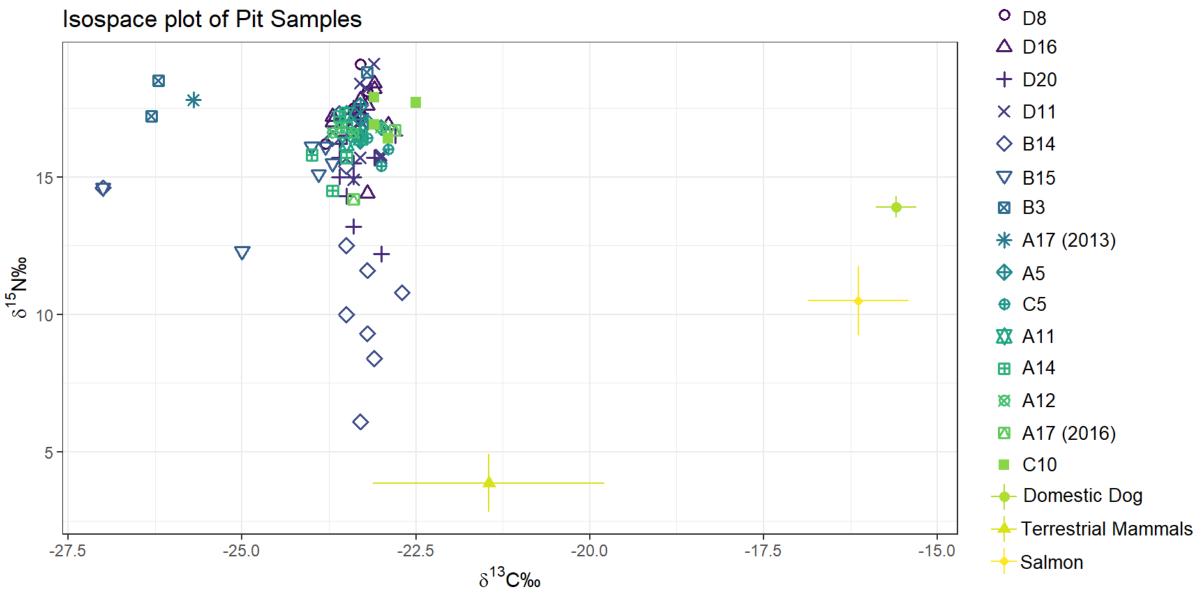

Figure 6. Isospace plot of pit feature values and faunal foodstuff categories

Running the SIMMR model with each feature as a separate group resulted in the majority of observations (pit feature values) outside the 50% interval boundary expected for a well-fitted model. This indicates that the model could not predict the contribution of each foodstuff group to the feature fill to a high degree. For each feature, the model generates summary measures including the means, standard deviations and credible intervals per foodstuff (an example of which can be seen in Table 3). Generally, a narrow credible interval for a foodstuff indicates the model is able to better distinguish the probable contribution of that foodstuff to the mixture while a wide-range in the credible interval indicates less certainty. Certainty is influenced by how distinguishable isotope values are between each input food source, where isotope values with more overlap are less distinguishable source inputs. This lack of certainty with the SIMMR model is most likely due to cumulative influence of additional anthropogenic factors and plant food sources influencing the final isotope values for the feature fill.

	2.5%	25%	50%	75%	97.5%
Domestic Dog	0.025	0.169	0.433	0.715	0.924
Terrestrial Mammals	0.019	0.094	0.230	0.547	0.910
Salmon	0.018	0.08	0.163	0.305	0.737

Table 3. Feature D8 Credible Intervals per Food Source

Even though the model's predictive power is fairly low, the SIMMR results do show some interesting trends across features that may be explored further with more robust foodsource isotope data in the future (Figure 7-9).

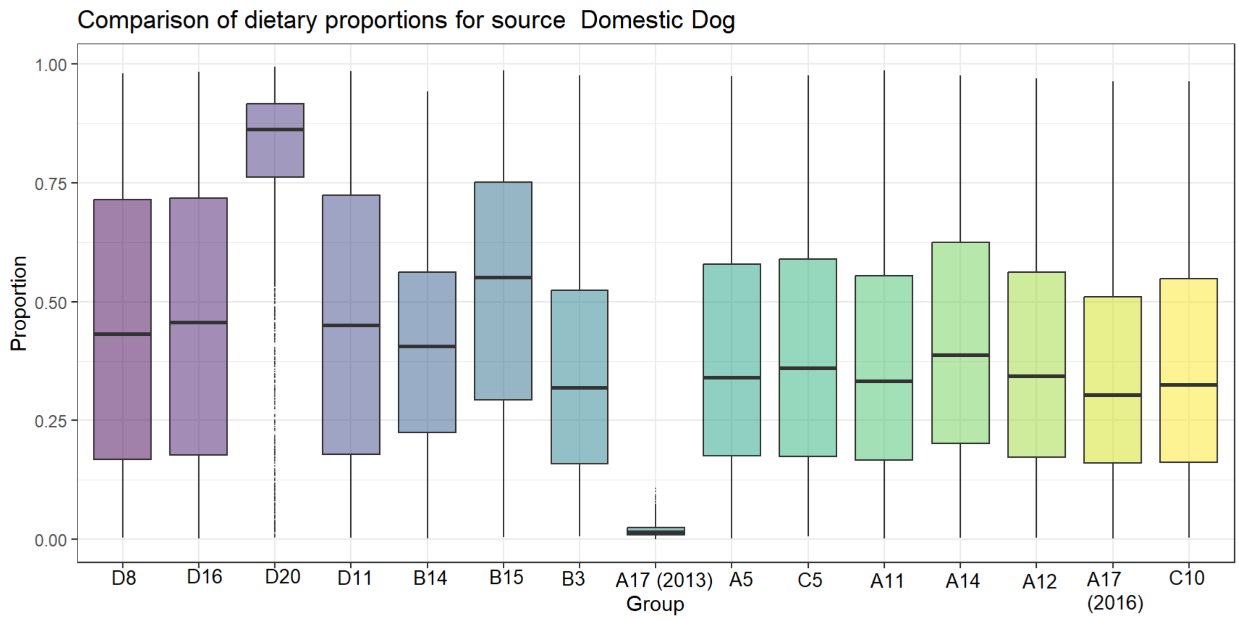

Figure 7. Comparison of Dietary Proportions for Domestic Dog by Feature for First Model Run

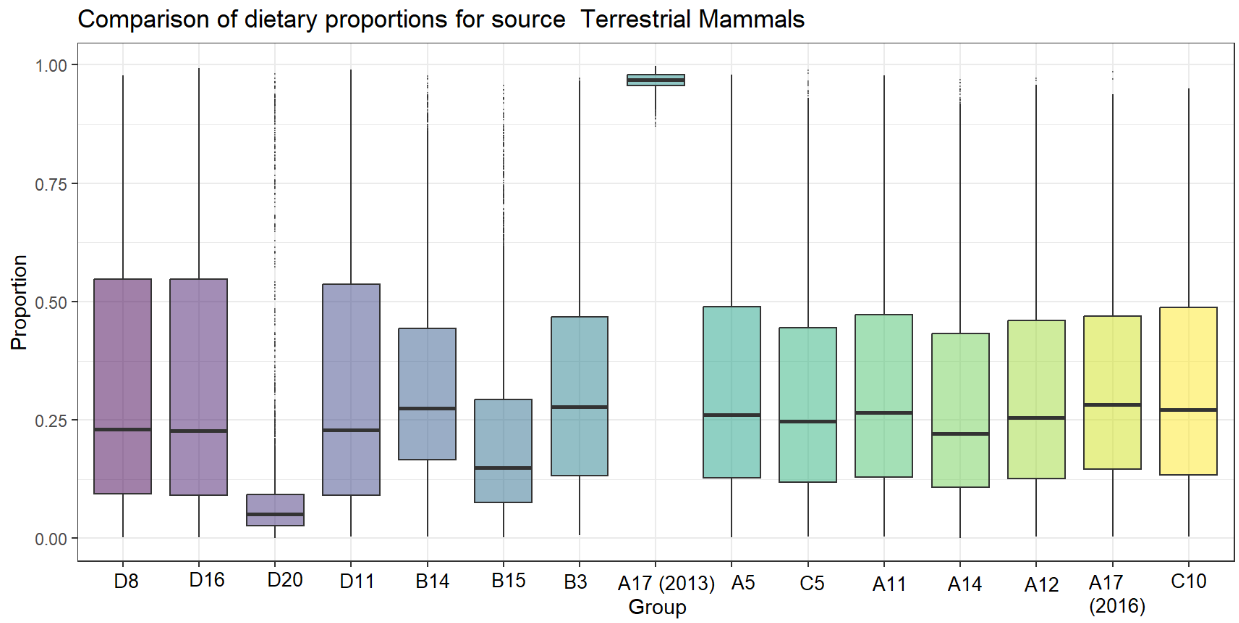

Figure 8. Comparison of Dietary Proportions for Terrestrial Mammal by Feature for First Model Run

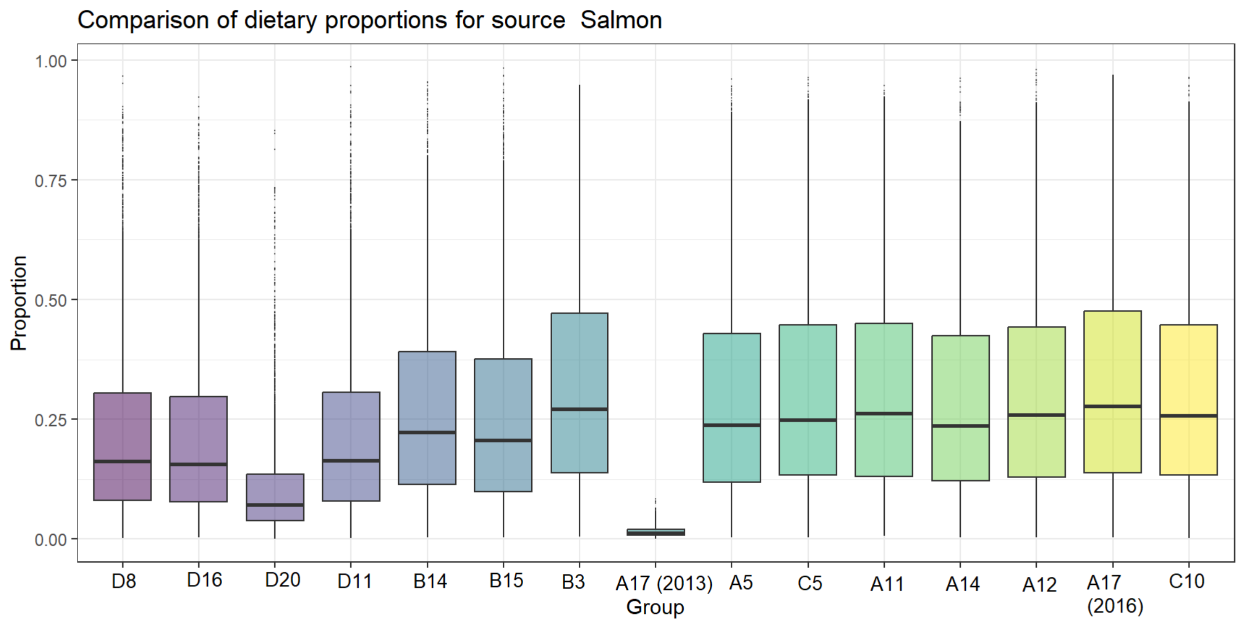

Figure 9. Comparison of Dietary Proportions for Salmon by Feature for First Model Run

To see if there were any distinctions between features that aligns with the faunal assemblage groupings and to account for the degree of isotopic overlap between salmon and dog, the dog food source was removed and the SIMMR modal was run again with just Terrestrial Mammal and Fish food sources (combining both Salmon and Other Fish). This model had similarly low levels of accuracy/predictive power for the contribution of foodstuffs to feature fill as the previous run (Figure 10).

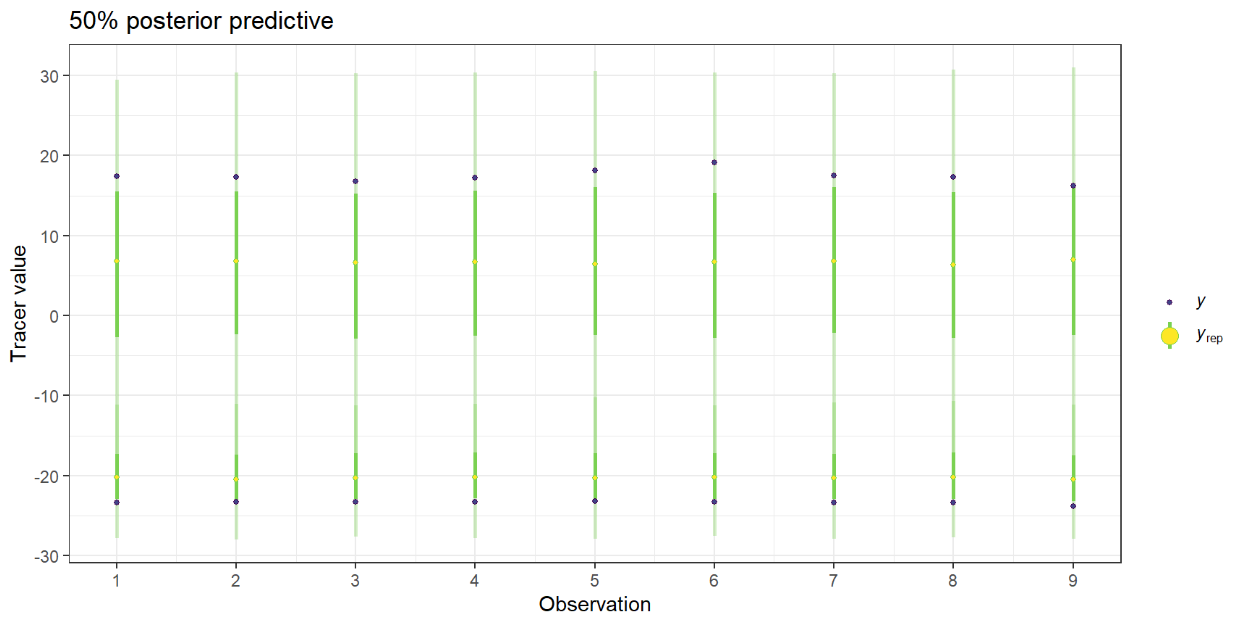

Figure 10. SIMMR Model accuracy for Terrestrial Mammals vs. Fish

For all features, the model predicts that terrestrial mammals may have had a slightly higher contributing proportion to the feature fill with some probabilities being somewhat higher than others (Table 4).

Feature	Faunal Assemblage Group	SIMMR Model Order by Highest to Lowest Proportion	SIMMR Model Probability
D8	2b	Terrestrial Mammals > Fish	0.744
D16	2b	Terrestrial Mammals > Fish	0.751
D20	2b	Terrestrial Mammals > Fish	0.804
D11	2b	Terrestrial Mammals > Fish	0.732
B14	N/A	Terrestrial Mammals > Fish	0.474
B15	N/A	Terrestrial Mammals > Fish	0.422
B3	2b	Terrestrial Mammals > Fish	0.475
A17 (2013)	1	Terrestrial Mammals > Fish	0
A5	1	Terrestrial Mammals > Fish	0.559
C5	2b	Terrestrial Mammals > Fish	0.517
A11	2b	Terrestrial Mammals > Fish	0.498
A14	2b	Terrestrial Mammals > Fish	0.501
A12	2b	Terrestrial Mammals > Fish	0.514
A17 (2016)	2a	Terrestrial Mammals > Fish	0.495
C10	1	Terrestrial Mammals > Fish	0.538

Table 4. Highest to Lowest Foodstuff Contribution to Feature Fill and Probability of Accuracy for Terrestrial Mammal and Fish Sources

Within the 2b Faunal Assemblage group, this run of the SIMMR model does seem to highlight potential distinctions between Features D8, D16, D20, and D11 compared to the rest of the pit features in considering the potential contribution of mammals and fish.

Results: Comparing Pit Features (Major and Trace Elements)

To potentially highlight differences in subsistence practices and foodstuffs between pit feature fill using element data, a selection of elements were compared. A majority of elements act as proxies for a multitude of different kinds of activity patterns and oftentimes overlap in the kinds of activities that leave behind enrichment of certain elements (Trant et al. 2024; Bintliff and Degryse 2022). Thus, distinguishing between fish or mammal preparation based solely on elemental composition is not possible. However, by comparing a suite of elements, it can reveal different degrees of elemental enrichment which can at least show how pit feature fill indicates activity variation – for instance, if all elemental markers are much lower or higher, then the fill may be showing high/low degrees of intensity of floor activity or high/low variation in population density comparative to the other pit feature fill.

Elements for comparison were selected if they were first observed in the majority of samples, then drawing on Trant et al (2024: 5-6), the element was selected if it related to foodstuff/midden/hearth activity. Elements used for comparison were Calcium (Ca), Potassium (K), and Phosphorus (P).

Drawing on the approach taken by Scott (2020: 594-595), element ppm values were standardized as a ratio to titanium measurements per sample since titanium values are reflective of lithogenic rather than anthropogenic processes. Both PPM and Ratio values for features can be found in **Dataset 2 Element Results**. Calcium element ratio to titanium values range from 3.378 to 9.258, potassium element ratio to titanium values range from 1.549 to 2.819, and phosphorus element ratio to titanium values range from 0.114 to 0.877.

Statistical tests were run without including the singular sample for Feature A17 (2013). Results of Ca, K, and P as ratios to Ti show statistically significant differences; for Ca (Kruskal–Wallis ANOVA, $\chi 2 = 29.099$, df = 13, $P < 0.006$), for K (Kruskal–Wallis ANOVA, $\chi 2 = 65.796$, df = 13, $P < 0.001$), and for P (Kruskal–Wallis ANOVA, $\chi 2 = 63.267$, df = 13, $P < 0.001$).

Based on pairwise comparison with Bonferroni adjustment, for Ca element values, Feature B15 is statistically significantly different from D16 and C5 (Figure 11).

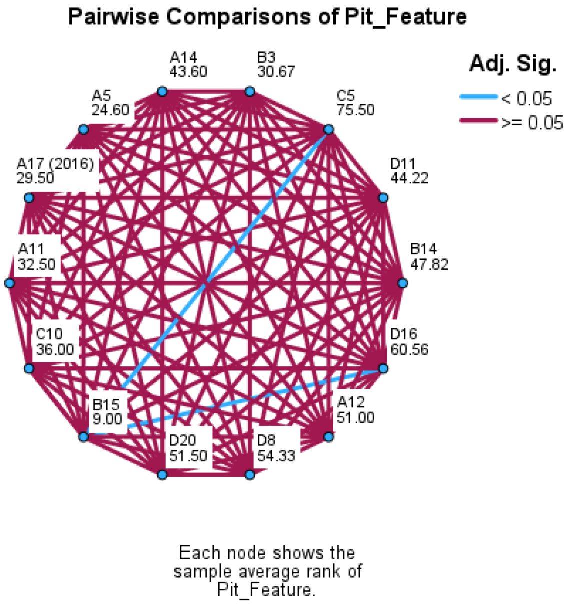

Figure 11. Kruskal-Wallis Test Pairwise Comparison for Ca Ratio values with Bonferroni adjustment

For K values, Feature C10 is statistically significantly different from A14, B14, and D20. Feature D20 is also different from B15 and D8, Feature D8 is different from A14, Feature D16 and D20 are different (Figure 12).

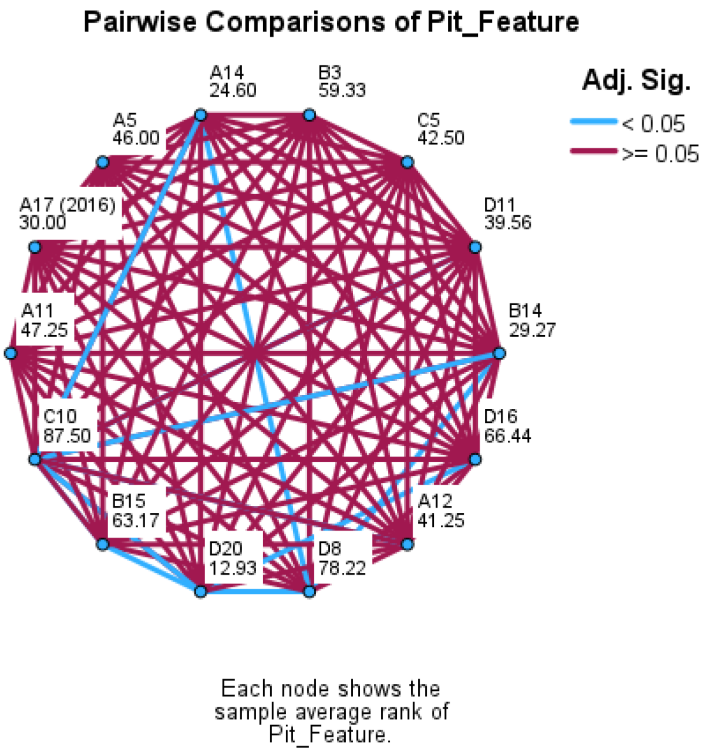

Figure 12. Kruskal-Wallis Test Pairwise Comparison for K Ratio values with Bonferroni adjustment

For P element values, Feature B14 is statistically significantly different from D11, A11, C10, D8, and A12. B15 is different from A11 and D16. C10 is different also from D20 (Figure 13).

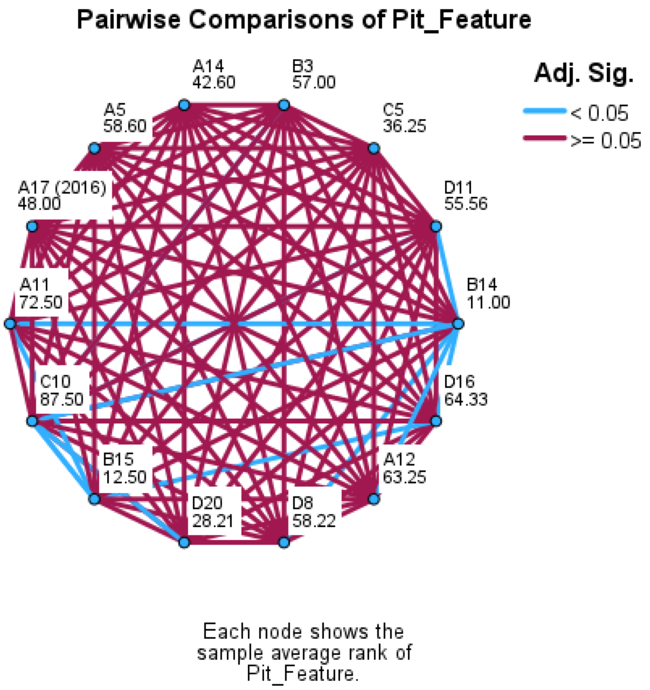

Figure 13. Kruskal-Wallis Test Pairwise Comparison for P Ratio values with Bonferroni adjustment

Kendall's rank tau correlation tests in R (using the cor.test function) were run between sample depth (level) and each element as a ratio to Ti. The test was run on a dataset that included Features B14 and B15 and a dataset that did not include those two features. For both datasets, persistent statistically significant correlations were found between calcium and sample depth and between phosphorus and sample depth.

Results for Ca ($\tau_b = 0.189$, $p = 0.014$) and for P ($\tau_b = -0.278$, $p = 0.0003$) indicate correlation with sample depth while K was uncorrelated to sample depth ($\tau_b = -0.126$, $p = 0.102$) when Features B14 and B15 were included. When Feature B14 and B15 were not included, correlations were reduced for Ca ($\tau_b = 0.164$, $p = 0.059$) and P ($\tau_b = -0.195$, $p = 0.025$) but still present.

Results: Comparing Based on Faunal Assemblage Types (Element Data)

Using Spearman's r correlation tests in R (using the cor.test function), no correlations were found between the average values of these three elements and faunal density (Table 5). Results were the following: for Ca averages to faunal density ($r_s = 0.339$, $p = 0.216$), for K averages to faunal density ($r_s = -0.225$, $p = 0.419$), and for P averages to faunal density ($r_s = 0.103$, $p = 0.714$).

Feature	Average Ca Ratio to Ti	Average K Ratio to Ti	Average P Ratio to Ti	Faunal Assemblage Type	Faunal Density
D8	6.00	2.248	0.336	2b	0.98

D16	6.484	2.084	0.409	2b	1.7
D11	5.792	1.870	0.363	2b	0.53
D20	6.200	1.677	0.221	2b	1.54
B3	5.181	2.039	0.326	2b	0.32
B15	4.315	2.060	0.167	2b	0.005
B14	5.944	1.826	0.157	2b	0.27
C5	7.026	1.905	0.250	2b	1.15
C10	5.340	2.675	0.855	1	2.58
A5	4.970	1.937	0.340	1	0.86
A11	5.214	1.932	0.398	2b	0.79
A12	6.153	1.899	0.372	2b	0.66
A14	5.611	1.801	0.282	2b	2.63
A17 (2013)	5.033	2.155	0.538	1	0.07
A17 (2016)	5.124	1.824	0.290	2a	2.69

Table 5. Summary Element and Faunal Density Data for Comparing Between Faunal Assemblage Types

References

Bintliff, J. and Degryse, P. A Review of Soil Geochemistry in Archaeology. *Journal of Archaeological Science: Reports* (2022) 43:103419.

Choy, K., Potter, B.A., McKinney, H.J., Reuther, J.D., Wang, S.W., and Wooller, M.J. Chemical Profiling of Ancient Hearths Reveals Recurrent Salmon Use in Ice Age Beringia. *PNAS* (2016) 113: 9757-9762.

Feddern, M.L., Holtgrieve, G.W., Perakis, S.S., Hart, J., Ro, H., Quinn, T.P. Riparian soil nitrogen cycling and isotopic enrichment in response to a long-term salmon carcass manipulation experiment. *Ecosphere* (2019) 10:1-16.

Goodale, N., Hill, K., Bailey, D.G., Nauman, W., Prentis, A.M., Rubinstein, E., Wegter, B., and Newlander, K. “Geochemical Characterization of Fur Trade Period Floor Sediments from Housepit 54, Bridge River Village.” In *The Last House at Bridge River: The Archaeology of an Aboriginal Household in British Columbia During the Fur Trade Period*, edited by Prentiss, A.M., Salt Lake City: The University of Utah Press (2016), p. 165-181.

Johnson, D.M., Hooper, P.R., and Conrey, R.M. XRF Analysis of Rocks and Minerals for Major and Trace Elements on a Single Low Dilution Li-tetraborate Fused Bead. *Advances in X-ray Analysis* (1999) 41:843-867.

Satterfield IV, F. R. and Finney, B.P. Stable Isotope Analysis of Pacific Salmon: Insights into Trophic Status and Oceanographic Conditions Over the Last 30 Years. *Progress in Oceanography* (2002) 53:231-246.

Scott, C. B. Integrating Multi-Scalar Sampling Strategies for Archaeological Sediment Chemistry. *Journal of Field Archaeology* (2020) 45:588-607.

Trant, P. L.K., Wouters, B., Croix, S., Sindbæk, S.M. Deckers, P., and Kristiansen, S.M. A multi-proxy geochemical and micromorphological study of the use of space and stratigraphy of a Viking-age house in Ribe, Denmark. *Archaeological and Anthropological Sciences* (2024) 16:1-35.

Food Source Isotope Data References:

Schwarcz, H. P., Chisholm, B.S., and Burchell, M. Isotopic Studies of the Diet of the People of the Coast of British Columbia. *American Journal of Physical Anthropology* (2014) 155:460-468.

Tifental, E. (2016) The Bridge River Dogs: Interpreting aDNA And Stable Isotope Analysis Collected From Dog Remains. Masters Thesis. Missoula (MT): The University of Montana.

Data:

Dataset 1-Isotope Results.

BagNum	Excavation Year	GeoChemLabNum	Block	Strat	Unit	Quad	FeatureNum	FeatLevel	δ13C (‰)	δ15N (‰)
428	2014	GC148	d	IIc	16	sw,se	d8	1	-23.4	17.4
408	2014	GC163	d	IIc	16	sw,se	d8	1	-23.3	17.3
402	2014	GC144	d	IIc	16	sw,se	d8	2	-23.3	16.8
404	2014	GC150	d	IIc	16	sw,se	d8	3	-23.3	17.2
407	2014	GC146	d	IIc	16	sw,se	d8	4	-23.2	18.1
403	2014	GC147	d	IIc	16	sw,se	d8	5	-23.3	19.1
401	2014	GC139	d	IIc	16	sw,se	d8	7	-23.4	17.5
430	2014	GC141	d	IIc	16	sw,se	d8	9	-23.4	17.3
431	2014	GC142	d	IIc	16	sw,se	d8	10	-23.8	16.2
55	2016	GC008	d	IId	8	sw	d16	1	-23.3	17.8
56	2016	GC014	d	IId	8	sw	d16	2	-23.1	18.2
73	2016	GC010	d	IId	8	sw	d16		-23.2	17.6
54	2016	GC015	d	IId	8	se	d16	1	-23.1	18.4
53	2016	GC009	d	IId	8	se	d16	2	-23.7	17.0
61	2016	GC013	d	IId	8	se	d16	3	-23.4	16.9
63	2016	GC012	d	IId	8	se	d16	4	-23.2	14.4
62	2016	GC011	d	IId	8	se	d16		-22.9	16.9
496	2016	GC158	d	IId	8	unk	d16	1	-23.7	17.2
121	2016	GC019	d	IIe	3	ne	d20	1	-23.4	13.2
126	2016	GC038	d	IIe	3	nw,ne	d20	1	-23.3	17.1
125	2016	GC040	d	IIe	3	nw,ne	d20	1	-23.6	15.7
130	2016	GC043	d	IIe	3	nw,ne	d20	1	-23.4	15.5

127	2016	GC037	d	IIe	3	nw,ne	d20	1	-23.6	15.0
129	2016	GC025	d	IIe	3	nw,ne	d20	1	-23.0	12.2
112	2016	GC022	d	IIe	3	ne	d20	3	-22.8	16.5
116	2016	GC032	d	IIe	3	nw,ne	d20	4	-23.5	16.5
114	2016	GC031	d	IIe	3	nw,ne	d20	5	-23.4	17.5
119	2016	GC041	d	IIe	3	nw	d20	6	-23.6	16.2
122	2016	GC042	d	IIe	3	nw	d20	7	-23.1	15.7
128	2016	GC133	d	IIe	3	nw	d20	8	-23.5	14.3
123	2016	GC039	d	IIe	3	nw	d20	4to9	-23.4	15.0
109	2016	GC033	d	IIe	3	ne	d20		-23.3	17.3
14	2016	GC020	d	IIe	7	ne	d11	1	-23.3	18.4
15	2016	GC028	d	IIe	11	se	d11	1	-23.1	19.1
22	2016	GC029	d	IIe	11	se	d11		-23.4	17.1
19	2016	GC018	d	IIe	11	se	d11	2	-23.0	15.7
17	2016	GC035	d	IIe	11	se	d11	3	-23.3	15.7
18	2016	GC036	d	IIe	12	se	d11	1	-23.2	18.2
21	2016	GC023	d	IIe	12	sw	d11		-23.0	15.8
16	2016	GC024	d	IIe	12	sw	d11	2	-23.4	14.9
20	2016	GC017	d	IIe	12	sw	d11		-23.4	16.8
300	2014	GC185	b	IIe	15	se	b14	2	-23.7	16.4
260	2014	GC175	b	IIe	15	sw	b14	3	-23.6	17.3
271	2014	GC180	b	IIe	11	ne	b14	4	-23.5	15.4
283	2014	GC182	b	IIe	11	ne	b14	5	-23.5	12.5
284	2014	GC183	b	IIe	11	ne	b14	6	-23.2	11.6
327	2014	GC186	b	IIe	15	se	b14	6	-23.2	9.3
347	2014	GC171	b	IIe	15	se	b14	7	-23.1	8.4
314	2014	GC172	b	IIe	15	se	b14	8	-23.3	6.1
383	2014	GC170	b	IIe	15	se	b14	9	-22.7	10.8

405	2014	GC187	b	IIe	15	se	b14	10	-23.5	10.0
406	2014	GC197	b	IIe	15	se	b14	11	-27.0	14.6
256	2014	GC169	b	IIe	6	ne	b15	1	-24.0	16.1
259	2014	GC177	b	IIe	6	ne	b15	1	-25.0	12.3
261	2014	GC176	b	IIe	6	ne	b15	1	-23.7	15.5
258	2014	GC174	b	IIe	6	ne	b15	1	-23.9	15.1
257	2014	GC173	b	IIe	6	ne	b15	1	-23.8	16.1
250	2014	GC196	b	IIe	6	ne	b15	2	-27.0	14.6
183	2014	GC199	b	IIe	9	se	b3	1	-26.2	18.5
116	2014	GC188	b	IIe	5	ne	b3	1	-23.2	18.8
182	2014	GC198	b	IIe	5	ne	b3	3	-26.3	17.2
429	2013	GC205	a	IIf	6	nw,sw	a17	3	-25.7	17.8
180	2014	GC229	a	IIh	14,15	ne,nw,ne	a5	1	-23.2	17.0
181	2014	GC231	a	IIh	14,15	se,sw,sw	a5	2	-23.3	16.3
179	2014	GC228	a	IIh	10,11	ne,nw,ne	a5	3	-23.3	17.1
185	2014	GC227	a	IIh	14,15	nw,ne.nw	a5	4	-23.0	16.8
206	2014	GC230	a	IIh	14,15	sw,se,sw	a5	5	-23.3	17.6
96	2016	GC089	c	IIk	7	ne	c5	1	-23.0	15.4
133	2016	GC087	c	IIk	7	nw	c5	1	-22.9	16.0
124	2016	GC068	c	IIk	7	nw	c5	1	-23.0	15.4
132	2016	GC077	c	IIk	7	nw	c5	3	-23.2	16.4
36	2016	GC092	a	IIl	10,14	nw,ne,sw,se	a11	1	-23.3	16.4
34	2016	GC099	a	IIl	10,14	nw,ne,sw,se	a11	1	-23.5	17.3

37	2016	GC097	a	IIl	10,14	nw,ne,sw,se	a11	3	-23.5	16.2
38	2016	GC100	a	IIl	10,14	nw,ne,sw,se	a11	4	-23.3	16.5
52	2016	GC107	a	IIm	7	sw,se	a14	1	-23.6	17.2
64	2016	GC119	a	IIm	7	sw,se	a14	2	-23.5	16.8
60	2016	GC105	a	IIm	7	sw,se	a14	3	-23.7	14.5
65	2016	GC118	a	IIm	7	sw,se	a14	4	-23.5	15.7
69	2016	GC123	a	IIm	7	sw,se	a14	5	-24.0	15.8
41	2016	GC103	a	IIm	14	ne	a12	1	-23.0	16.8
42	2016	GC116	a	IIm	14	se	a12	1	-23.6	16.8
46	2016	GC122	a	IIm	14	se	a12	2	-23.7	16.6
85	2016	GC108	a	IIm	10	se	a12	3	-23.4	16.6
45	2016	GC109	a	IIn	14,15	nw,ne,sw,se	a17	2	-22.8	16.7
83	2016	GC110	a	IIn	15	nw,ne,sw,se	a17	3	-23.4	14.2
31	2022	GC31	c	IIh	8	nw	c10	2	-23.141	16.8906
35	2022	GC35	c	IIh	8	nw	c10	3	-23.1	17.9062
42	2022	GC42	c	IIh	8	nw	c10	4	-22.939	16.3761
48	2022	GC48	c	IIh	8	nw	c10	5	-22.5	17.7089

Dataset 2-Element Results

**Bag Num**	**Excavation Year**	**GeoChem LabNum**	**Block**	**Strat**	**Unit**	**Quad**	**FeatureNum**	**Feat Level**	**Ti PPM**	**Ca PPM**	**K PPM**	**P PPM**	**Ca Ratio to Ti**	**K Ratio to Ti**	**P Ratio to To**
428	2014	GC148	d	IIc	16	sw,se	d8	1	5716.223	32577.46	12863.33	1829.036	5.699	2.250	0.320

408	2014	GC163	d	IIc	16	sw,se	d8	1	6251.576	38294.34	13112.38	2293.387	6.126	2.097	0.367
402	2014	GC144	d	IIc	16	sw,se	d8	2	6180.235	32553.16	12728.84	1773.611	5.267	2.060	0.287
404	2014	GC150	d	IIc	16	sw,se	d8	3	5749.795	31593.31	12402.59	1743.061	5.495	2.157	0.303
407	2014	GC146	d	IIc	16	sw,se	d8	4	5825.931	35912.25	13718.39	2135.403	6.164	2.355	0.367
403	2014	GC147	d	IIc	16	sw,se	d8	5	6010.577	36313.91	14231.43	2219.196	6.042	2.368	0.369
401	2014	GC139	d	IIc	16	sw,se	d8	7	5972.809	36019.45	13729.19	2007.096	6.031	2.299	0.336
430	2014	GC141	d	IIc	16	sw,se	d8	9	5695.84	32617.48	12899.03	1774.047	5.727	2.265	0.311
431	2014	GC142	d	IIc	16	sw,se	d8	10	4494.444	33698.11	10738.12	1634.393	7.498	2.389	0.364
55	2016	GC008	d	IId	8	sw	d16	1	6303.132	41221.04	12396.78	3073.27	6.540	1.967	0.488
56	2016	GC014	d	IId	8	sw	d16	2	5797.755	36506.16	11834.76	2891.719	6.297	2.041	0.499
73	2016	GC010	d	IId	8	sw	d16		5312.76	35138.23	11335	2249.309	6.614	2.134	0.423
54	2016	GC015	d	IId	8	se	d16	1	6297.137	58298.79	12523.79	3884.574	9.258	1.989	0.617
53	2016	GC009	d	IId	8	se	d16	2	5578.938	35786.46	12276.41	2922.268	6.415	2.200	0.524
61	2016	GC013	d	IId	8	se	d16	3	5555.557	30934.36	11295.99	1805.47	5.568	2.033	0.325
63	2016	GC012	d	IId	8	se	d16	4	5291.778	37220.86	10481.6	1183.135	7.034	1.981	0.224
62	2016	GC011	d	IId	8	se	d16		5036.991	29544.98	11151.54	1791.068	5.866	2.214	0.356

496	2016	GC158	d	IId	8	unk	d16	1	6091.509	29058.27	13434.48	1395.235	4.770	2.205	0.229
121	2016	GC019	d	IIe	3	ne	d20	1	5190.462	24905.15	8039.269	846.2184	4.798	1.549	0.163
126	2016	GC038	d	IIe	3	nw,ne	d20	1	5095.742	29125.45	8442.727	1432.767	5.716	1.657	0.281
125	2016	GC040	d	IIe	3	nw,ne	d20	1	4842.153	32779	9129.27	1177.025	6.770	1.885	0.243
130	2016	GC043	d	IIe	3	nw,ne	d20	1	5256.407	32487.4	8276.695	1186.626	6.181	1.575	0.226
127	2016	GC037	d	IIe	3	nw,ne	d20	1	4942.27	36473.29	7956.253	1161.314	7.380	1.610	0.235
129	2016	GC025	d	IIe	3	nw,ne	d20	1	4763.619	41890	8260.922	776.3912	8.794	1.734	0.163
112	2016	GC022	d	IIe	3	ne	d20	3	6074.723	23513.63	9587.518	1145.603	3.871	1.578	0.189
116	2016	GC032	d	IIe	3	nw,ne	d20	4	5419.471	25077.39	9103.535	1069.229	4.627	1.680	0.197
114	2016	GC031	d	IIe	3	nw,ne	d20	5	5548.963	31785.57	8924.22	1503.903	5.728	1.608	0.271
119	2016	GC041	d	IIe	3	nw	d20	6	5125.117	35544.18	9361.714	1255.144	6.935	1.827	0.245
122	2016	GC042	d	IIe	3	nw	d20	7	5210.246	31559.72	8414.502	1131.201	6.057	1.615	0.217
128	2016	GC133	d	IIe	3	nw	d20	8	5245.017	39567.22	9529.407	1163.496	7.544	1.817	0.222
123	2016	GC039	d	IIe	3	nw	d20	4to9	5404.483	35855.78	8149.681	1038.68	6.634	1.508	0.192
109	2016	GC033	d	IIe	3	ne	d20		6082.517	24243.34	10126.29	1218.048	3.986	1.665	0.200
14	2016	GC020	d	IIe	7	ne	d11	1	5280.987	43418.74	10499.86	3046.648	8.222	1.988	0.577

15	2016	GC028	d	IIe	11	se	d11	1	5376.907	38971.88	10679.18	3111.675	7.248	1.986	0.579
22	2016	GC029	d	IIe	11	se	d11		5176.074	27388.73	9849.018	1751.79	5.291	1.903	0.338
19	2016	GC018	d	IIe	11	se	d11	2	5785.165	23665.15	9067.008	1293.112	4.091	1.567	0.224
17	2016	GC035	d	IIe	11	se	d11	3	5468.03	25762.79	8873.58	1414.437	4.712	1.623	0.259
18	2016	GC036	d	IIe	12	se	d11	1	4846.35	33078.46	10608.61	2437.842	6.825	2.189	0.503
21	2016	GC023	d	IIe	12	sw	d11		5183.868	31211.66	9810.831	1284.82	6.021	1.893	0.248
16	2016	GC024	d	IIe	12	sw	d11	2	5533.975	22466.59	9085.271	1220.23	4.060	1.642	0.220
20	2016	GC017	d	IIe	12	sw	d11		5087.948	28810.99	10416.85	1659.705	5.663	2.047	0.326
300	2014	GC185	b	IIe	15	se	b14	2	6167.646	20837.08	10909.96	810.4319	3.378	1.769	0.131
260	2014	GC175	b	IIe	15	sw	b14	3	5274.992	24903.01	10964.75	1232.887	4.721	2.079	0.234
271	2014	GC180	b	IIe	11	ne	b14	4	5863.1	27962.64	11589.03	1029.515	4.769	1.977	0.176
283	2014	GC182	b	IIe	11	ne	b14	5	5797.755	27599.57	11102.56	1061.373	4.760	1.915	0.183
284	2014	GC183	b	IIe	11	ne	b14	6	5538.172	31995.69	9926.223	890.2968	5.777	1.792	0.161
327	2014	GC186	b	IIe	15	se	b14	6	5396.09	35847.92	9711.212	685.1794	6.643	1.800	0.127
347	2014	GC171	b	IIe	15	se	b14	7	4936.874	36556.19	8778.942	585.6756	7.405	1.778	0.119
314	2014	GC172	b	IIe	15	se	b14	8	5171.278	38672.42	9045.423	590.0398	7.478	1.749	0.114

383	2014	GC170	b	IIe	15	se	b14	9	4946.466	42155.86	8487.556	728.385	8.522	1.716	0.147
405	2014	GC187	b	IIe	15	se	b14	10	5509.995	30942.94	9567.594	710.4918	5.616	1.736	0.129
406	2014	GC197	b	IIe	15	se	b14	11	6033.358	38148.54	10726.5	1251.653	6.323	1.778	0.207
256	2014	GC169	b	IIe	6	ne	b15	1	5940.436	28557.27	12550.36	1169.606	4.807	2.113	0.197
259	2014	GC177	b	IIe	6	ne	b15	1	5520.786	21390.97	10715.71	717.0381	3.875	1.941	0.130
261	2014	GC176	b	IIe	6	ne	b15	1	5828.929	24632.14	12159.35	932.6295	4.226	2.086	0.160
258	2014	GC174	b	IIe	6	ne	b15	1	5520.786	24807.95	11702.77	986.7456	4.494	2.120	0.179
257	2014	GC173	b	IIe	6	ne	b15	1	5689.246	26453.19	12100.41	1063.556	4.650	2.127	0.187
250	2014	GC196	b	IIe	6	ne	b15	2	5963.816	22896.84	11766.69	918.6641	3.839	1.973	0.154
183	2014	GC199	b	IIe	9	se	b3	1	6186.83	34720.13	12014.91	2259.346	5.612	1.942	0.365
116	2014	GC188	b	IIe	5	ne	b3	1	5744.999	28089.14	12586.06	2047.683	4.889	2.191	0.356
182	2014	GC198	b	IIe	5	ne	b3	3	5496.806	27719.64	10911.62	1416.183	5.043	1.985	0.258
429	2013	GC205	a	IIf	6	nw,sw	a17	3	5873.891	29565.71	12658.28	3161.426	5.033	2.155	0.538
180	2014	GC229	a	IIh	14,15	ne,nw,ne	a5	1	5956.622	28189.2	11638.84	1851.73	4.732	1.954	0.311
181	2014	GC231	a	IIh	14,15	se,sw,sw	a5	2	5556.756	27102.14	10480.77	1810.27	4.877	1.886	0.326

179	2014	GC228	a	IIh	10,11	ne,nw,ne	a5	3	5667.664	29300.56	11051.09	1944.251	5.170	1.950	0.343
185	2014	GC227	a	IIh	14,15	nw,ne.nw	a5	4	5892.476	28424.33	11369.04	2029.789	4.824	1.929	0.344
206	2014	GC230	a	IIh	14,15	sw,se,sw	a5	5	5373.909	28215.64	10566.28	2032.408	5.250	1.966	0.378
96	2016	GC089	c	IIk	7	ne	c5	1	5406.881	34336.33	10372.02	1362.94	6.350	1.918	0.252
133	2016	GC087	c	IIk	7	nw	c5	1	5414.075	37671.84	10111.35	1287.875	6.958	1.868	0.238
124	2016	GC068	c	IIk	7	nw	c5	1	5042.386	38678.13	9192.362	961.4333	7.671	1.823	0.191
132	2016	GC077	c	IIk	7	nw	c5	3	5363.718	38230.73	10802.87	1709.894	7.128	2.014	0.319
36	2016	GC092	a	IIl	10,14	nw,ne,sw,se	a11	1	5767.18	29230.52	10860.98	2392.891	5.068	1.883	0.415
34	2016	GC099	a	IIl	10,14	nw,ne,sw,se	a11	1	5408.08	29061.85	9991.806	2096.998	5.374	1.848	0.388
37	2016	GC097	a	IIl	10,14	nw,ne,sw,se	a11	3	5345.733	26577.55	10727.33	1936.832	4.972	2.007	0.362
38	2016	GC100	a	IIl	10,14	nw,ne,sw,se	a11	4	4966.849	27036.39	9891.356	2134.967	5.443	1.991	0.430
52	2016	GC107	a	IIm	7	sw,se	a14	1	6450.01	30488.39	11507.68	2076.486	4.727	1.784	0.322
64	2016	GC119	a	IIm	7	sw,se	a14	2	6419.435	32579.6	12050.6	2276.803	5.075	1.877	0.355

60	2016	GC105	a	IIm	7	sw,se	a14	3	6000.985	35476.28	10665.07	1063.556	5.912	1.777	0.177
65	2016	GC118	a	IIm	7	sw,se	a14	4	5824.732	35316.19	10427.64	1149.094	6.063	1.790	0.197
69	2016	GC123	a	IIm	7	sw,se	a14	5	6108.895	38370.81	10875.93	2195.193	6.281	1.780	0.359
41	2016	GC103	a	IIm	14	ne	a12	1	5608.913	32945.53	11139.92	2807.926	5.874	1.986	0.501
42	2016	GC116	a	IIm	14	se	a12	1	5575.94	29161.19	10640.16	1869.623	5.230	1.908	0.335
46	2016	GC122	a	IIm	14	se	a12	2	6075.922	31726.25	11611.45	2385.908	5.222	1.911	0.393
85	2016	GC108	a	IIm	10	se	a12	3	6294.14	52151.66	11282.7	1646.176	8.286	1.793	0.262
45	2016	GC109	a	IIn	14,15	nw,ne,sw,se	a17	2	6027.363	31602.6	11180.59	2213.522	5.243	1.855	0.367
83	2016	GC110	a	IIn	15	nw,ne,sw,se	a17	3	5671.261	28387.88	10168.63	1213.684	5.006	1.793	0.214
31	2022	GC31	c	IIh	8	nw	c10	2	5290.277	27114.07	14911.77	4640.031	5.125	2.819	0.877
35	2022	GC35	c	IIh	8	nw	c10	3	5237.042	27078.06	14039.17	4423.639	5.170	2.681	0.845
42	2022	GC42	c	IIh	8	nw	c10	4	5214.228	28001.5	13763.94	4535.382	5.370	2.640	0.870
48	2022	GC48	c	IIh	8	nw	c10	5	5147.353	29329.98	13182.44	4270.057	5.698	2.561	0.830

Dataset 3-Isotope Results Outliers Removed

BagNum	Excavation Year	GeoChemLabNum	Block	Strat	Unit	Quad	FeatureNum	FeatLevel	δ13C (‰)	δ15N (‰)

428	2014	GC148	d	IIc	16	sw,se	d8	1	-23.4	17.4
408	2014	GC163	d	IIc	16	sw,se	d8	1	-23.3	17.3
402	2014	GC144	d	IIc	16	sw,se	d8	2	-23.3	16.8
404	2014	GC150	d	IIc	16	sw,se	d8	3	-23.3	17.2
401	2014	GC139	d	IIc	16	sw,se	d8	7	-23.4	17.5
430	2014	GC141	d	IIc	16	sw,se	d8	9	-23.4	17.3
55	2016	GC008	d	IId	8	sw	d16	1	-23.3	17.8
56	2016	GC014	d	IId	8	sw	d16	2	-23.1	18.2
73	2016	GC010	d	IId	8	sw	d16		-23.2	17.6
54	2016	GC015	d	IId	8	se	d16	1	-23.1	18.4
53	2016	GC009	d	IId	8	se	d16	2	-23.7	17.0
61	2016	GC013	d	IId	8	se	d16	3	-23.4	16.9
62	2016	GC011	d	IId	8	se	d16		-22.9	16.9
496	2016	GC158	d	IId	8	unk	d16	1	-23.7	17.2
121	2016	GC019	d	IIe	3	ne	d20	1	-23.4	13.2
126	2016	GC038	d	IIe	3	nw,ne	d20	1	-23.3	17.1
125	2016	GC040	d	IIe	3	nw,ne	d20	1	-23.6	15.7
130	2016	GC043	d	IIe	3	nw,ne	d20	1	-23.4	15.5
127	2016	GC037	d	IIe	3	nw,ne	d20	1	-23.6	15.0
129	2016	GC025	d	IIe	3	nw,ne	d20	1	-23.0	12.2
116	2016	GC032	d	IIe	3	nw,ne	d20	4	-23.5	16.5
114	2016	GC031	d	IIe	3	nw,ne	d20	5	-23.4	17.5
119	2016	GC041	d	IIe	3	nw	d20	6	-23.6	16.2
122	2016	GC042	d	IIe	3	nw	d20	7	-23.1	15.7
128	2016	GC133	d	IIe	3	nw	d20	8	-23.5	14.3
123	2016	GC039	d	IIe	3	nw	d20	4to9	-23.4	15.0
109	2016	GC033	d	IIe	3	ne	d20		-23.3	17.3
14	2016	GC020	d	IIe	7	ne	d11	1	-23.3	18.4

15	2016	GC028	d	IIe	11	se	d11	1	-23.1	19.1
22	2016	GC029	d	IIe	11	se	d11		-23.4	17.1
19	2016	GC018	d	IIe	11	se	d11	2	-23.0	15.7
17	2016	GC035	d	IIe	11	se	d11	3	-23.3	15.7
18	2016	GC036	d	IIe	12	se	d11	1	-23.2	18.2
21	2016	GC023	d	IIe	12	sw	d11		-23.0	15.8
16	2016	GC024	d	IIe	12	sw	d11	2	-23.4	14.9
20	2016	GC017	d	IIe	12	sw	d11		-23.4	16.8
300	2014	GC185	b	IIe	15	se	b14	2	-23.7	16.4
260	2014	GC175	b	IIe	15	sw	b14	3	-23.6	17.3
271	2014	GC180	b	IIe	11	ne	b14	4	-23.5	15.4
283	2014	GC182	b	IIe	11	ne	b14	5	-23.5	12.5
284	2014	GC183	b	IIe	11	ne	b14	6	-23.2	11.6
327	2014	GC186	b	IIe	15	se	b14	6	-23.2	9.3
347	2014	GC171	b	IIe	15	se	b14	7	-23.1	8.4
314	2014	GC172	b	IIe	15	se	b14	8	-23.3	6.1
383	2014	GC170	b	IIe	15	se	b14	9	-22.7	10.8
405	2014	GC187	b	IIe	15	se	b14	10	-23.5	10.0
256	2014	GC169	b	IIe	6	ne	b15	1	-24.0	16.1
261	2014	GC176	b	IIe	6	ne	b15	1	-23.7	15.5
258	2014	GC174	b	IIe	6	ne	b15	1	-23.9	15.1
257	2014	GC173	b	IIe	6	ne	b15	1	-23.8	16.1
183	2014	GC199	b	IIe	9	se	b3	1	-26.2	18.5
116	2014	GC188	b	IIe	5	ne	b3	1	-23.2	18.8
182	2014	GC198	b	IIe	5	ne	b3	3	-26.3	17.2
180	2014	GC229	a	IIh	14,15	ne,nw,ne	a5	1	-23.2	17.0
179	2014	GC228	a	IIh	10,11	ne,nw,ne	a5	3	-23.3	17.1

185	2014	GC227	a	IIh	14,15	nw,ne.nw	a5	4	-23.0	16.8
96	2016	GC089	c	IIk	7	ne	c5	1	-23.0	15.4
133	2016	GC087	c	IIk	7	nw	c5	1	-22.9	16.0
124	2016	GC068	c	IIk	7	nw	c5	1	-23.0	15.4
132	2016	GC077	c	IIk	7	nw	c5	3	-23.2	16.4
36	2016	GC092	a	IIl	10,14	nw,ne,sw,se	a11	1	-23.3	16.4
34	2016	GC099	a	IIl	10,14	nw,ne,sw,se	a11	1	-23.5	17.3
37	2016	GC097	a	IIl	10,14	nw,ne,sw,se	a11	3	-23.5	16.2
38	2016	GC100	a	IIl	10,14	nw,ne,sw,se	a11	4	-23.3	16.5
52	2016	GC107	a	IIm	7	sw,se	a14	1	-23.6	17.2
64	2016	GC119	a	IIm	7	sw,se	a14	2	-23.5	16.8
60	2016	GC105	a	IIm	7	sw,se	a14	3	-23.7	14.5
65	2016	GC118	a	IIm	7	sw,se	a14	4	-23.5	15.7
69	2016	GC123	a	IIm	7	sw,se	a14	5	-24.0	15.8
41	2016	GC103	a	IIm	14	ne	a12	1	-23.0	16.8
42	2016	GC116	a	IIm	14	se	a12	1	-23.6	16.8
46	2016	GC122	a	IIm	14	se	a12	2	-23.7	16.6
85	2016	GC108	a	IIm	10	se	a12	3	-23.4	16.6
45	2016	GC109	a	IIn	14,15	nw,ne,sw,se	a17	2	-22.8	16.7
83	2016	GC110	a	IIn	15	nw,ne,sw,se	a17	3	-23.4	14.2
31	2022	GC31	c	IIh	8	nw	c10	2	-23.141	16.8906
35	2022	GC35	c	IIh	8	nw	c10	3	-23.1	17.9062
42	2022	GC42	c	IIh	8	nw	c10	4	-22.939	16.3761

48	2022	GC48	c	IIh	8	nw	c10	5	-22.5	17.7089

Dataset 4-Comparative Isotopes

Common Name	Species	$\delta^{13}C$	$\delta^{15}N$	Food Group	Reference
Beaver	Castor canadensis	-23.10	4.90	Terrestrial Mammals	Schwarcz et al 2014*
Beaver	Castor canadensis	-23.20	6.30	Terrestrial Mammals	Schwarcz et al 2014*
Beaver	Castor canadensis	-20.71	5.33	Terrestrial Mammals	Tifental 2016
Beaver	Castor canadensis	-20.92	3.52	Terrestrial Mammals	Tifental 2016
Elk	Cervus canadiensis	-23.20	2.80	Terrestrial Mammals	Schwarcz et al 2014*
Elk	Cervus canadiensis	-23.70	4.40	Terrestrial Mammals	Schwarcz et al 2014*
Moose	Alces alces	-23.20	2.10	Terrestrial Mammals	Schwarcz et al 2014*
Moose	Alces alces	-23.20	3.00	Terrestrial Mammals	Schwarcz et al 2014*
Mule deer	O. hemionus	-19.78	3.67	Terrestrial Mammals	Tifental 2016
Mule deer	O. hemionus	-20.43	4.17	Terrestrial Mammals	Tifental 2016
Mule deer	O. hemionus	-20.07	4.04	Terrestrial Mammals	Tifental 2016
Mule deer	O. hemionus	-21.46	2.04	Terrestrial Mammals	Tifental 2016

Mule deer	O. hemionus	-21.74	3.61	Terrestrial Mammals	Tifental 2016
Mule deer	O. hemionus	-21.22	3.09	Terrestrial Mammals	Tifental 2016
Mule deer	O. hemionus	-20.61	4.02	Terrestrial Mammals	Tifental 2016
Mule deer	O. hemionus	-20.40	3.52	Terrestrial Mammals	Tifental 2016
Mule deer	O. hemionus	-20.82	5.88	Terrestrial Mammals	Tifental 2016
Mule deer	O. hemionus	-19.42	4.80	Terrestrial Mammals	Tifental 2016
Mule deer	O. hemionus	-20.58	2.60	Terrestrial Mammals	Tifental 2016
Mule deer	O. hemionus	-20.52	3.59	Terrestrial Mammals	Tifental 2016
Mule deer	O. hemionus	-20.56	3.03	Terrestrial Mammals	Tifental 2016
Mule deer	O. hemionus	-20.73	4.07	Terrestrial Mammals	Tifental 2016
Mule deer	O. hemionus	-19.30	4.14	Terrestrial Mammals	Tifental 2016
Mule deer	O. hemionus	-20.28	3.03	Terrestrial Mammals	Tifental 2016
Mule deer	O. hemionus	-20.51	3.99	Terrestrial Mammals	Tifental 2016
Mule deer	O. hemionus	-20.5	4.12	Terrestrial Mammals	Tifental 2016
Deer	Odocoileus sp.	-23.10	2.40	Terrestrial Mammals	Schwarcz et al 2014*
Deer	Odocoileus sp.	-23.40	4.20	Terrestrial Mammals	Schwarcz et al 2014*

Deer	Odocoileus sp.	-26.30	3.00	Terrestrial Mammals	Schwarcz et al 2014*
Deer	Odocoileus sp.	-23.90	4.60	Terrestrial Mammals	Schwarcz et al 2014*
Bighorn Sheep	O. canadensis	-20.28	4.14	Terrestrial Mammals	Tifental 2016
Bighorn Sheep	O. canadensis	-19.24	5.77	Terrestrial Mammals	Tifental 2016
Salmon	Oncorhynchus	-16.57	9.61	Salmon	Tifental 2016
Sockeye	O. nerka	-16.27	9.69	Salmon	Tifental 2016
Sockeye	O. nerka	-16.14	9.70	Salmon	Tifental 2017
Sockeye	O. nerka	-16.70	11.00	Salmon	Tifental 2018
Sockeye	O. nerka	-17.03	9.66	Salmon	Tifental 2019
Sockeye	O. nerka	-16.06	9.99	Salmon	Tifental 2020
Sockeye	O. nerka	-14.68	13.22	Salmon	Tifental 2021
Sockeye	O. nerka	-15.66	11.08	Salmon	Tifental 2022
Trout	Onchorhyncus mykis	-21.70	11.40	Trout	Schwarcz et al 2014*
Trout	Onchorhyncus mykis	-20.80	14.70	Trout	Schwarcz et al 2014*
Domestic Dog	C. familiaris	-15.47	13.76	Dogs	Tifental 2016
Domestic Dog	C. familiaris	-15.39	14.51	Dogs	Tifental 2016

Domestic Dog	C. familiaris	-15.88	13.53	Dogs	Tifental 2016
Domestic Dog	C. familiaris	-15.86	13.47	Dogs	Tifental 2016
Domestic Dog	C. familiaris	-15.91	13.75	Dogs	Tifental 2016
Domestic Dog	C. familiaris	-15.47	14.14	Dogs	Tifental 2016
Domestic Dog	C. familiaris	-15.16	14.23	Dogs	Tifental 2016

* δ13C values for modern day species samples adjusted by 1.5‰ to account for the Suess effect from the original value (marked by * on Reference)

Dataset 5 – Isotope Food Source Averages and Standard Deviations (Based on Dataset 4 Values)

Species Group	$\delta^{13}C$ Average	$\delta^{13}C$ Standard Deviation	$\delta^{15}N$ Average	$\delta^{15}N$ Standard Deviation
Domestic Dog	-15.591	0.292	13.912	0.387
Terrestrial Mammals	-21.449	1.666	3.870	1.051
Salmon	-16.138	0.724	10.493	1.255
Fish (Both Salmon and Other Fish)	-17.161	2.257	11.005	1.730
